# Clinical impact of broad- versus narrow-spectrum empiric therapy in acute cholangitis: A Japanese claims database study

**DOI:** 10.1371/journal.pone.0346452

**Published:** 2026-04-02

**Authors:** Kazuhiro Aoto, Ryo Inose, Yuichi Muraki

**Affiliations:** 1 Laboratory of Clinical Pharmacoepidemiology, Kyoto Pharmaceutical University, 5 Misasaginakauchicho, Kyoto Yamashina-ku, Kyoto, Japan; 2 Department of Pharmacy, University Hospital, Kyoto Prefectural University of Medicine, Kajii-Cho, Kawaramachi-Hirokoji, Kamigyo-Ku, Kyoto, Japan; University of Illinois Urbana-Champaign College of Veterinary Medicine, UNITED STATES OF AMERICA

## Abstract

The clinical benefit of broad-spectrum empiric therapy in patients with acute cholangitis is unclear. We aimed to evaluate the impact of broad-spectrum and narrow-spectrum empiric therapies on patient outcomes using a Japanese claims database. The study included patients who were diagnosed with acute cholangitis between April 2014 and August 2022, aged 18–99 years, received antibiotics, had blood cultures collected, and underwent biliary drainage. Patients who received empiric therapy with carbapenems, piperacillin/tazobactam, or fourth-generation cephalosporins were included in the broad-spectrum group, whereas others were included in the narrow-spectrum group. Of the 4,755 eligible patients, 3,377 were categorized into the narrow-spectrum group and 1,378 into the broad-spectrum group. In the multivariate logistic regression analysis, older age, higher Charlson Comorbidity Index, the presence of sepsis, and intensive care unit admission were associated with increased 30-day in-hospital mortality, whereas the receipt of broad-spectrum empiric therapy was not (adjusted odds ratio, 1.37 [95% confidence interval {CI}, 0.84–2.23]). In the propensity score matching analysis, there was also no association between the receipt of broad-spectrum empiric therapy and 30-day in-hospital mortality (odds ratio, 1.43 [95% CI, 0.82–2.50]). Furthermore, in the propensity score-matched cohort, the broad-spectrum group had longer duration of intravenous antibiotic therapy (median interquartile range [IQR]: 8 [6 –11] day vs. 9 [7 –13] day; difference 1 day [95% CI, 0.31–1.69 day]) and length of hospital stay (median [IQR]: 13 [9 –20] day vs. 16 [11 –25] day; difference 3 day [95% CI, 1.87–4.13 day]), compared with the narrow-spectrum group. In this large-scale study using a Japanese claims database, broad-spectrum empiric therapy was not associated with improved clinical outcomes, compared with narrow-spectrum empiric therapy. Therefore, the necessity of broad-spectrum empiric therapy may be limited in clinical practice, and narrow-spectrum empiric therapy may represent an effective treatment strategy for acute cholangitis.

## Introduction

Acute cholangitis is frequently complicated by bacteremia, and some patients develop septic shock or organ dysfunction [1–4]. Antimicrobial therapy and biliary drainage are the key treatments for acute cholangitis that improve patient outcomes [5].

The major pathogens that cause acute cholangitis are *Escherichia coli* and *Klebsiella pneumoniae* [1]. Over the recent decades, antimicrobial resistance among these *Enterobacteriales* has become a global public health concern [6–8]. To address the potential presence of these antimicrobial resistance, clinical guidelines for acute cholangitis recommend empiric therapy with carbapenems, piperacillin/tazobactam, or fourth-generation cephalosporins [9,10]. These broad-spectrum antibiotics are also positioned as empiric therapy targeting *Pseudomonas aeruginosa* [9,10], which is rarely isolated as a causative pathogen of acute cholangitis [1].

The use of broad-spectrum antibiotics in empiric therapy is a common treatment strategy for infections; however, recent clinical reports have highlighted their overuse [11–13]. Such overuse not only contributes to the spread of antimicrobial resistance but may also be associated with higher mortality, an increased risk of *Clostridioides difficile* infection (CDI), and prolonged length of hospital stay (LOS) [11,14–17]. Consequently, the consistent benefits of broad-spectrum empiric therapy on patient outcomes have been questioned, and the overuse of broad-spectrum antibiotics in empiric therapy has been recognized as an issue that needs to be addressed [11,16–18].

To date, it remains unclear whether broad-spectrum empiric therapy is associated with better outcomes than narrow-spectrum empiric therapy in acute cholangitis. A retrospective cohort study showed that patient outcomes did not improve with broad-spectrum empiric therapy compared to narrow-spectrum empiric therapy, suggesting that the benefit of broad-spectrum empiric therapy is limited [19]. However, this was a single-center study with a small sample size. To our knowledge, no large-scale cohort study has evaluated the benefits of broad-spectrum versus narrow-spectrum empiric therapy on clinical outcomes in patients with acute cholangitis.

Healthcare databases enable comprehensive data collection from large patient populations across multiple facilities and have therefore been widely used to evaluate medication efficacy [20]. Particularly, claims databases have recently been used for studies on antimicrobial therapy in large populations [17,21].

This study aimed to examine whether broad-spectrum empiric therapy is associated with improved 30-day in-hospital mortality compared with narrow-spectrum empiric therapy using a Japanese claims database. Furthermore, as a secondary objective, the impact of broad-spectrum empiric therapy on the incidence of CDI, duration of intravenous (IV) antibiotic therapy, and LOS was evaluated.

## Materials and methods

### Study design and data source

This retrospective cohort study was conducted using a hospital database provided by JMDC, Inc. (Tokyo, Japan) [22]. The database contains claims data for all patients who visited approximately 500 hospitals. This represents approximately 7% of all hospitals in Japan and contains data on approximately 17 million patients. The database includes information on patient demographics, diagnoses, prescriptions, and procedures.

Data extraction from the database began on January 23, 2025. In this study, we used only anonymized administrative claims database and did not have access to information that could identify individual patients. This study was confirmed by the ethics committee of Kyoto Pharmaceutical University as it did not require an ethical review, on January 22, 2025 (NR-00009). This study followed the Reporting of Studies Conducted using Observational Routinely-Collected Data for Pharmacoepidemiology reporting guidelines [23] (S1 Checklist).

### Population

We enrolled patients diagnosed with acute cholangitis between April 2014 and August 2022. The database used in this study contained only monthly diagnostic data and did not include detailed daily diagnostic information. Therefore, to enhance the reliability of the acute cholangitis diagnosis, the following inclusion criteria were adopted: age 18–99 years, first episode of acute cholangitis, receiving antibiotics listed in the Tokyo Guidelines 2018 for more than 2 days [10], blood culture obtained on the day of antibiotic initiation, and biliary drainage performed within 2 days of antibiotic initiation. Patients with unknown discharge dates were excluded as their outcomes could not be assessed.

### Exposures and outcomes

The eligible patients were categorized into broad-spectrum and narrow-spectrum groups. The broad-spectrum group included patients who received carbapenems, piperacillin/tazobactam, or fourth-generation cephalosporins, whereas all others were included in the narrow-spectrum group (S1 Table). The primary outcome was the 30-day in-hospital mortality. The secondary outcomes included the incidence of CDI during hospitalization, duration of IV antibiotic therapy, and LOS. CDI was evaluated as a representative adverse event associated with antimicrobial therapy [24]. The proportion of patients in each group who received broad-spectrum antibiotics as definitive therapy was also investigated.

### Collected data and definitions

The database used in this study did not contain information on culture results. Therefore, antibiotic therapy initiated on the day of blood culture collection was considered empiric therapy, and the date was defined as the index date. The following data were collected based on the index date: age; sex; acquisition type (community-acquired or healthcare-associated); Charlson Comorbidity Index (CCI) [25]; presence of sepsis; vasopressor prescription; intensive care unit (ICU) admission; concomitant prescription of vancomycin, history of immunosuppressant or antibiotic prescription within 90 days; hospital bed count; treatment year. Community-acquired cholangitis was defined as cases in which empiric therapy was initiated within 2 days of admission, whereas healthcare-associated cholangitis was defined as cases in which empiric therapy was initiated more than 2 days after admission [26]. The CCI was collected as a measure of comorbidity burden to assess the impact of comorbidities on prognosis [27].

Diagnoses were identified using the International Classification of Diseases, 10th edition codes. Antibiotics were identified using anatomical therapeutic chemical codes, and blood culture collection was identified using the Japanese procedure code. Definitive therapy was defined as the antibiotic administered on the day of completion of IV antibiotic therapy. All codes used for collection in this study are summarized in S1 and S2 Tables.

### Statistical analysis

Univariate and multivariate logistic regression analyses were performed to evaluate the association between receipt of broad-spectrum empiric therapy and 30-day in-hospital mortality. The exposure variable was the receipt of broad-spectrum empiric therapy (yes/no), and the outcome was 30-day in-hospital mortality (yes/no). Similar to a previous study [28], the multivariable model was adjusted for all variables with a p value ≤ 0.1 in the univariable analysis. Multicollinearity among covariates was assessed by calculating the variance inflation factor for each variable, with values less than 10 considered to indicate no concerning multicollinearity [29]. The association between receipt of broad-spectrum empiric therapy and 30-day in-hospital mortality was also evaluated using a conditional logistic regression model after propensity score (PS) matching. PS matching analysis was performed to adjust for the differences in baseline characteristics between the groups [30]. A nearest neighbor 1:1 matching with a maximum caliper of 0.2 times the standard deviation was applied in the PS matching. The PS was estimated by logistic regression using the following variables: age, sex, acquisition type, CCI, presence of sepsis, vasopressor prescription, ICU admission, history of immunosuppressant or antibiotic prescription within 90 days, hospital bed count, and treatment year. After PS matching, the balance of variables between the groups was evaluated using absolute standardized differences, with differences < 0.1 interpreted as well balanced.

The incidence of CDI, duration of IV antibiotic therapy, and LOS were reported as between-group differences after PS matching; bootstrapping with 1,000 samples was used for 95% confidence intervals (CIs). Categorical variables were expressed as absolute numbers, and continuous variables were expressed as medians and interquartile ranges (IQRs). Significance in all statistical analyses was defined as a two-sided p value < 0.05. All statistical analyses were performed using the Stata software (version 18.0; StataCorp LLC., College Station, TX, USA).

### Sensitivity analysis

Sensitivity analyses were performed to evaluate the robustness of the primary results. First, to rule out the possibility that blood cultures were not collected on the day of antimicrobial therapy initiation in severely ill patients, we examined the baseline characteristics and 30-day in-hospital mortality rates in patients without blood culture collection. Second, a sensitivity analysis restricted to patients with sepsis in the PS-matched cohort was conducted to assess the robustness of the association between broad-spectrum empiric therapy and 30-day in-hospital mortality.

## Results

### Patient characteristics

**Fig 1:**
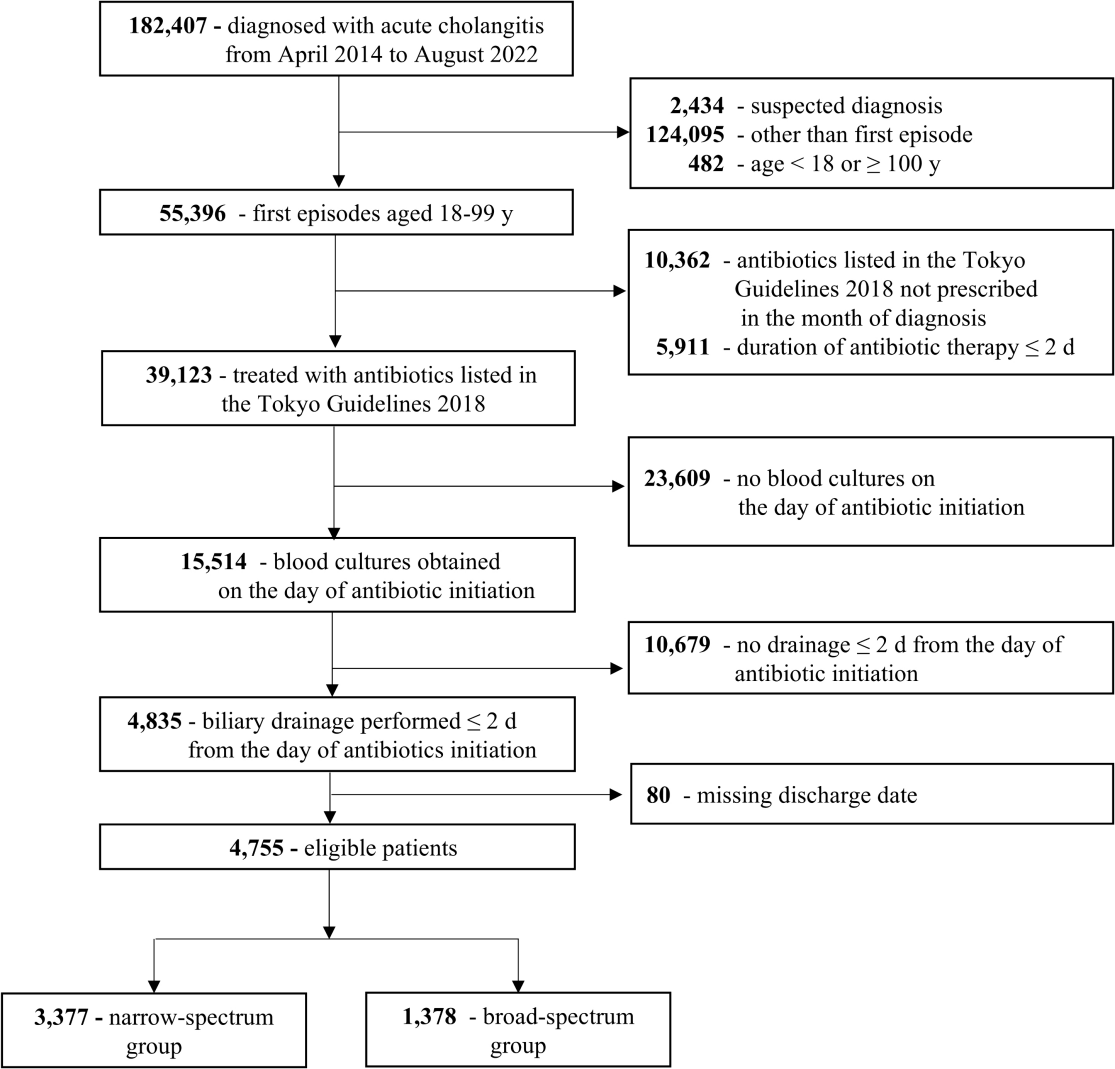
Flow diagram of the patient selection process.

The study cohort included 182,407 patients diagnosed with acute cholangitis (Fig 1). Of these, 4,755 patients were eligible, with 3,377 categorized into the narrow-spectrum group and 1,378 into the broad-spectrum group. Table 1 shows the baseline characteristics of the patients in each group, and Table 2 lists the antibiotics included in each group. A larger proportion of patients with sepsis was found in the broad-spectrum group (n = 489 [35.5%]) than in the narrow-spectrum group (n = 743 [22.0%]) (Table 1).

**Table 1:** Baseline characteristics of the patients.

	Narrow-spectrum group (n = 3377)	Broad-spectrum group (n = 1378)
**Demographics**		
**Male sex**	1864 (55.2)	753 (54.6)
**Age, y, median (IQR)**	81 (73–87)	82 (75–88)
**Age ≥ 75 y**	2418 (71.6)	1058 (76.8)
**Community-acquired cholangitis**	3187 (94.4)	1254 (91.0)
**CCI, median (IQR)**	1 (0–2)	1 (0–2)
**Sepsis**	743 (22.0)	489 (35.5)
**Vasopressor prescription**	162 (4.8)	87 (6.3)
**ICU admission**	106 (3.1)	106 (7.7)
**History of prescriptions**		
Immunosuppressant(s)	259 (7.7)	161 (11.7)
Antibiotic(s)	401 (11.9)	201 (14.6)
**Combination antibiotics**		
Vancomycin	6 (0.2)	6 (0.4)
**Hospital bed count**		
≤ 199	221 (6.5)	109 (7.9)
200–499	1957 (58.0)	668 (48.5)
≥ 500	1199 (35.5)	601 (43.6)
**Treatment year**		
2014	80 (2.4)	23 (1.7)
2015	138 (4.1)	34 (2.5)
2016	167 (4.9)	57 (4.1)
2017	220 (6.5)	70 (5.1)
2018	499 (14.8)	218 (15.8)
2019	590 (17.5)	211 (15.3)
2020	656 (19.4)	267 (19.4)
2021	699 (20.7)	317 (23.0)
2022	328 (9.7)	181 (13.1)

Data are presented as numbers (%) unless otherwise indicated.

Abbreviations: CCI, Charlson Comorbidity Index; ICU, intensive care unit; IQR, interquartile range.

**Table 2:** Antibiotics included in the narrow-spectrum and broad-spectrum groups.

Group	Classification	Antibiotics	n (%)
**Narrow-spectrum group**			**3377 (100)**
	Combinations of penicillins and beta-lactamase inhibitors	Ampicillin/sulbactam	463 (13.71)
	First-generation cephalosporins	Cefazolin	12 (0.36)
	Second-generation cephalosporins	Cefmetazole	663 (19.63)
		Cefotiam	37 (1.10)
		Flomoxef	7 (0.21)
	Third-generation cephalosporins	Cefoperazone/sulbactam	2011 (59.55)
		Ceftriaxone	147 (4.35)
		Cefotaxime	9 (0.27)
		Ceftazidime	4 (0.12)
	Fluoroquinolones	Ciprofloxacin	2 (0.06)
		Levofloxacin	21 (0.62)
	Monobactams	Aztreonam	1 (0.03)
**Broad-spectrum group**			**1378 (100)**
	Fourth-generation cephalosporins	Cefepime	14 (1.02)
		Cefozopran	13 (0.94)
	Combinations of penicillins and beta-lactamase inhibitors	Piperacillin/tazobactam	813 (59.00)
	Carbapenems	Meropenem	465 (33.74)
		Doripenem	62 (4.50)
		Imipenem/cilastatin	11 (0.80)

Cefoperazone/sulbactam (n = 2,011 [59.6%]) was the most commonly administered antibiotic in the narrow-spectrum group, followed by cefmetazole (n = 663 [19.6%]) (Table 2). In contrast, piperacillin/tazobactam (n = 813 [59.0%]) was the most common antibiotic in the broad-spectrum group, followed by meropenem (n = 465 [33.7%]). The 30-day in-hospital mortality rates in the narrow- and broad-spectrum groups were 1.3% (n = 43) and 2.2% (n = 31), respectively. Supporting Information S1 File shows the results of a sensitivity analysis conducted in patients without blood culture collection.

### Logistic regression analysis of variables associated with 30-day in-hospital mortality

Table 3 shows the variables associated with 30-day in-hospital mortality in the logistic regression analysis. In multivariate logistic regression analysis, older age (adjusted odds ratio [aOR], 1.06 [95% CI, 1.03–1.09], p < 0.001), higher CCI (aOR, 1.32 [95% CI, 1.19–1.47], p < 0.001), the presence of sepsis (aOR, 1.89 [95% CI, 1.17–3.06], p = 0.010), and ICU admission (aOR, 2.65 [95% CI, 1.26–5.58], p = 0.010) were significantly associated with increased 30-day in-hospital mortality, whereas receipt of broad-spectrum empiric therapy was not significantly (aOR, 1.37 [95% CI, 0.84–2.23], p = 0.20). Variance inflation factor values ranged from 1.01 to 1.17, indicating no concerning collinearity of the variables.

**Table 3:** Univariate and multivariate logistic analyses of variables associated with 30-day in-hospital mortality.

Variables	Univariate analysis		Multivariate analysis	
	OR (95% CI)	p value^a^	aOR (95% CI)	p value^a^
**Broad-spectrum empiric therapy**	1.78 (1.12–2.84)	0.015	1.37 (0.84–2.23)	0.20
**Male sex**	0.81 (0.51–1.29)	0.38		
**Age, y**	1.04 (1.01–1.07)	0.002	1.06 (1.03–1.09)	< 0.001
**Community-acquired cholangitis**	0.50 (0.25–1.02)	0.057	0.59 (0.29–1.22)	0.16
**CCI**	1.33 (1.22–1.46)	< 0.001	1.32 (1.19–1.47)	< 0.001
**Sepsis**	2.21 (1.39–3.52)	0.001	1.89 (1.17–3.06)	0.010
**Vasopressor prescription**	0.50 (0.12–2.04)	0.33		
**ICU admission**	3.05 (1.50–6.22)	0.002	2.65 (1.26–5.58)	0.010
**History of immunosuppressant prescription**	2.68 (1.51–4.77)	0.001	1.64 (0.84–3.21)	0.15
**History of antibiotic prescription**	2.25 (1.32–3.86)	0.003	1.59 (0.87–2.92)	0.13

Abbreviations: aOR, adjusted odds ratio; CCI, Charlson Comorbidity Index; CI, confidence interval; ICU, intensive care unit; OR, odds ratio.

^a^Significance level: p = 0.05.

### Patient outcomes in the PS-matched cohort

After PS matching, 1,378 patients in each group were included, and all baseline variables were well-balanced (S3 Table). The antibiotics included were similar to those before the PS matching analysis (S4 Table). A conditional logistic regression model showed that receipt of broad-spectrum empiric therapy was not significantly associated with 30-day in-hospital mortality (OR, 1.43 [95% CI, 0.82–2.50], p = 0.21). Furthermore, no significant association was observed even in a sensitivity analysis restricted to patients with sepsis (OR, 1.48 [95% CI, 0.71–3.12], p = 0.30).

Table 4 presents the results regarding the incidence of CDI during hospitalization, duration of IV antibiotic therapy, and LOS. The incidence of CDI did not significantly differ between the narrow-and broad-spectrum groups (5 [0.36%] vs. 8 [0.58%], difference [95% CI]: 0.22 [−0.32–0.75]%, p = 0.43). In contrast, compared with the narrow-spectrum group, the broad-spectrum group had longer duration of IV antibiotic therapy (median [IQR]: 8 [6 –11] day vs. 9 [7 –13] day; difference [95% CI]: 1 [0.31–1.69] day, p = 0.004) and LOS (median [IQR]: 13 [9 –20] day vs. 16 [11 –25] day; difference [95% CI]: 3 [1.87–4.13], p < 0.001).

**Table 4:** Incidence of CDI, duration of IV antibiotic therapy, and LOS in the PS-matched cohort.

Outcomes	No. of events (%) or median (IQR)		Difference, % or days (95% CI)	p value^e^
	Narrow-spectrum group (n = 1378)	Broad-spectrum group (n = 1378)		
**CDI**	5 (0.36)^a^	8 (0.58)^a^	0.22 (−0.32–0.75)^b^	0.43
**Duration of IV antibiotic therapy**	8 (6–11)^c^	9 (7–13)^c^	1 (0.31–1.69)^d^	0.004
**LOS**	13 (9–20)^c^	16 (11–25)^c^	3 (1.87–4.13)^d^	< 0.001

Abbreviations: CDI, Clostridioides difficile infection; CI, confidence interval; IQR, interquartile range; IV, intravenous; LOS, length of hospital stay.

^a^Data are presented as numbers (%).

^b^Data are presented as % (95% CI).

^c^Data are presented as median (IQR).

^d^Data are presented as days (95% CI).

^e^Significance level: p = 0.05.

As definitive therapy, 87.3% (n = 1,203) of the patients in the narrow-spectrum group continued receiving narrow-spectrum antibiotics, and only 10.7% (n = 147) required escalation to broad-spectrum antibiotics (S1 Fig). In contrast, in the broad-spectrum group, although 34.8% (n = 480) of the patients were de-escalated to narrow-spectrum antibiotics, 63.2% (n = 871) continued to receive broad-spectrum antibiotics.

## Discussion

This study showed that administration of broad-spectrum empiric therapy was not associated with improved 30-day in-hospital mortality. This finding was consistent across both the logistic regression and PS matching analyses. Furthermore, sensitivity analysis restricted to patients with sepsis showed similar results, supporting the robustness of the results. In addition, in the PS-matched cohort, patients in the broad-spectrum group had longer duration of IV antibiotic therapy and LOS (Table 4). Collectively, the present study did not identify any clear clinical benefits of broad-spectrum empiric therapy. To the best of our knowledge, this is the largest study to evaluate the impact of broad-spectrum empiric therapy for acute cholangitis.

Piperacillin/tazobactam and meropenem, which are generally active against third-generation cephalosporin-resistant *Enterobacterales* and *P. aeruginosa* [31–33], were commonly used in broad-spectrum empiric therapy (Table 2). Although blood culture results were unavailable in this study, the prevalence of third-generation cephalosporin resistance among *E. coli* and *K. pneumoniae*, the main pathogens of acute cholangitis [1], is approximately 10–20% in Japan [32]. In addition, the isolation rate of *P. aeruginosa* in acute cholangitis is low, at approximately 1–2% [1]. These epidemiological data suggest that a large proportion of patients with acute cholangitis in Japan can be treated without broad-spectrum antibiotics. Indeed, this study showed that escalation to broad-spectrum antibiotics as definitive therapy was uncommon in the narrow-spectrum group, suggesting that most patients were adequately treated with narrow-spectrum antibiotics (S1 Fig).

In contrast, despite the lower likelihood of resistant pathogens in Japan, 63.2% of the patients in the broad-spectrum group continued to receive broad-spectrum antibiotics without de-escalation. This finding implies that de-escalation may not be sufficiently implemented in patients with acute cholangitis who received broad-spectrum empiric therapy. A previous study indicated that de-escalation was less likely when culture results were negative [34]. Considering that the positive blood culture rate for acute cholangitis is approximately 40% [1], some patients in the broad-spectrum group may not have undergone de-escalation because their blood cultures were negative. Furthermore, the continuation of broad-spectrum antibiotics may be influenced by physicians’ strong sense of reassurance regarding the efficacy of these agents, as has been reported in studies on antibiotic prescription behavior [35,36].

This study also showed that the duration of IV antibiotic therapy and LOS were longer in the broad-spectrum group versus the narrow-spectrum group (Table 4). Our previous single-center study showed that narrow-spectrum empiric therapy is associated with shorter IV antibiotic therapy and LOS, through active switching to oral antibiotics [19]. These findings suggest that measures are needed to avoid unnecessary prolonged use of antibiotics, particularly in patients who have received broad-spectrum empiric therapy. Although broad-spectrum empiric therapy has been reported to be associated with an increased incidence of CDI [11,16], no significant difference was observed between the groups in this study (Table 4). This result was probably because the incidence was lower than that reported in previous studies [11,16].

Nevertheless, de-escalation and the duration of IV antibiotic therapy may be influenced not only by the physician's psychology but also by patient-related factors, including individual clinical background [37,38]. Although this study adjusted for patient background using PS matching analysis, it could not fully account for these factors. Therefore, our findings do not imply that broad-spectrum empiric therapy is always unnecessary for the treatment of acute cholangitis. Rather, these results suggest that broad-spectrum empiric therapy for acute cholangitis could be an important focus of antimicrobial stewardship, highlighting the need for more judicious indications.

This study has some limitations. First, to address the confounders, we performed multivariate logistic regression and PS matching analyses. However, disease severity may not have been adequately adjusted for because the database did not contain patients’ vital parameters, laboratory results, and detailed organ dysfunction measures. In addition, information regarding the diagnosis and treatment at institutions outside the database could not be collected and was unknown. Therefore, residual confounding may exist based on the indications and severity. Second, only patients who had undergone biliary drainage were included in this study to enhance the reliability of the acute cholangitis diagnosis. Consequently, our cohort may have included a higher proportion of patients with favorable prognoses. Early and successful source control via drainage may offset the mortality-reducing effects of broad-spectrum empiric therapy, limiting its generalizability to situations in which drainage is delayed or unavailable. Additionally, it was unclear whether source control was successfully achieved after drainage. Differences in the achievement of source control may have influenced patient outcomes in both groups. Third, our findings may have been influenced by the low prevalence of antimicrobial resistance in Japan. Broad-spectrum empiric therapy may be beneficial for patient outcomes in regions with a high prevalence of antimicrobial resistance. Further research is warranted to evaluate the impact of broad-spectrum empiric therapy in such settings. Despite these limitations, this study provides new insights into the role of broad-spectrum antibiotics in empiric therapy for acute cholangitis.

## Conclusion

In this study, broad-spectrum empiric therapy was not associated with improved clinical outcomes compared with narrow-spectrum empiric therapy. This finding, based on large-scale Japanese claims data, suggests that the necessity of broad-spectrum empiric therapy is limited and that narrow-spectrum empiric therapy may represent an effective treatment strategy for acute cholangitis. The active use of narrow-spectrum antibiotics as empiric therapy can help reduce the use of broad-spectrum antibiotics and may contribute to the prevention of antimicrobial resistance.

## Supporting information

S1 Checklist The reporting of studies conducted using observational routinely-collected data for pharmacoepidemiology checklist. (PDF)

S1 Table Antibiotics included in the analysis. (DOCX)

S2 Table Codes used for variable definitions. (DOCX)

S1 File Supporting information. A sensitivity analysis in the patient selection process. (DOCX)

S3 Table Baseline characteristics of the patients before and after PS matching. (DOCX)

S4 Table Antibiotics included in the narrow-spectrum and broad-spectrum groups after PS matching. (DOCX)

S1 Fig Proportions of antibiotics in the categories used as definitive therapy stratified by empiric therapy. (PDF)

## References

[1] Gomi H, Takada T, Hwang T-L, Akazawa K, Mori R, Endo I, et al. Updated comprehensive epidemiology, microbiology, and outcomes among patients with acute cholangitis. J Hepatobiliary Pancreat Sci. 2017;24(6):310–8. doi: 10.1002/jhbp.452 28371094

[2] Lavillegrand J-R, Mercier-Des-Rochettes E, Baron E, Pène F, Contou D, Favory R, et al. Acute cholangitis in intensive care units: clinical, biological, microbiological spectrum and risk factors for mortality: a multicenter study. Crit Care. 2021;25(1):49. doi: 10.1186/s13054-021-03480-1 33549136 PMC7866656

[3] Lee C-C, Chang I-J, Lai Y-C, Chen S-Y, Chen S-C. Epidemiology and prognostic determinants of patients with bacteremic cholecystitis or cholangitis. Am J Gastroenterol. 2007;102(3):563–9. doi: 10.1111/j.1572-0241.2007.01095.x 17335448

[4] Otani T, Ichiba T, Seo K, Naito H. Blood cultures should be collected for acute cholangitis regardless of severity. J Infect Chemother. 2022;28(2):181–6. doi: 10.1016/j.jiac.2021.10.004 34635451

[5] Miura F, Okamoto K, Takada T, Strasberg SM, Asbun HJ, Pitt HA, et al. Tokyo guidelines 2018: initial management of acute biliary infection and flowchart for acute cholangitis. J Hepatobiliary Pancreat Sci. 2018;25(1):31–40. doi: 10.1002/jhbp.509 28941329

[6] Paterson DL, Bonomo RA. Extended-spectrum beta-lactamases: a clinical update. Clin Microbiol Rev. 2005;18(4):657–86. doi: 10.1128/CMR.18.4.657-686 16223952 PMC1265908

[7] Pitout JDD, Laupland KB. Extended-spectrum beta-lactamase-producing Enterobacteriaceae: an emerging public-health concern. Lancet Infect Dis. 2008;8(3):159–66. doi: 10.1016/S1473-3099(08)70041-0 18291338

[8] Castanheira M, Deshpande LM, Mendes RE, Canton R, Sader HS, Jones RN. Variations in the occurrence of resistance phenotypes and carbapenemase genes among enterobacteriaceae isolates in 20 years of the SENTRY antimicrobial surveillance program. Open Forum Infect Dis. 2019;6(Suppl 1):S23–33. doi: 10.1093/ofid/ofy347 30895212 PMC6419900

[9] Solomkin JS, Mazuski JE, Bradley JS, Rodvold KA, Goldstein EJC, Baron EJ, et al. Diagnosis and management of complicated intra-abdominal infection in adults and children: guidelines by the Surgical infection society and the infectious diseases society of America. Clin Infect Dis. 2010;50(2):133–64. doi: 10.1086/649554 20034345

[10] Gomi H, Solomkin JS, Schlossberg D, Okamoto K, Takada T, Strasberg SM. Tokyo guidelines 2018: antimicrobial therapy for acute cholangitis and cholecystitis. J Hepatobil Pancreatic Sci. 2018;25:3–16. doi: 10.1002/jhbp.518 29090866

[11] Rhee C, Kadri SS, Dekker JP, Danner RL, Chen H-C, Fram D, et al. Prevalence of antibiotic-resistant pathogens in culture-proven sepsis and outcomes associated with inadequate and broad-spectrum empiric antibiotic use. JAMA Netw Open. 2020;3(4):e202899. doi: 10.1001/jamanetworkopen.2020.2899 32297949 PMC7163409

[12] Goodman KE, Baghdadi JD, Magder LS, Heil EL, Sutherland M, Dillon R, et al. Patterns, Predictors, and Intercenter Variability in Empiric Gram-Negative Antibiotic Use Across 928 United States Hospitals. Clin Infect Dis. 2023;76(3):e1224–35. doi: 10.1093/cid/ciac504 35737945 PMC9907550

[13] Rhee C, Chen T, Kadri SS, Lawandi A, Yek C, Walker M, et al. Trends in empiric broad-spectrum antibiotic use for suspected community-onset sepsis in US hospitals. JAMA Netw Open. 2024;7(6):e2418923. doi: 10.1001/jamanetworkopen.2024.18923 38935374 PMC11211962

[14] Hsueh P-R, Chen W-H, Luh K-T. Relationships between antimicrobial use and antimicrobial resistance in Gram-negative bacteria causing nosocomial infections from 1991-2003 at a university hospital in Taiwan. Int J Antimicrob Agents. 2005;26(6):463–72. doi: 10.1016/j.ijantimicag.2005.08.016 16280243 PMC7126312

[15] Kagami K, Ishiguro N, Iwasaki S, Taki K, Fukumoto T, Hayasaka K, et al. Correlation between antibiotic use and resistance of gram-negative bacteria at a university hospital in Japan from 2013 to 2021: a study using the Japan Surveillance for Infection Prevention and Healthcare Epidemiology (J-SIPHE) system. Eur J Hosp Pharm. 2023;ejhpharm-2023-003797. doi: 10.1136/ejhpharm-2023-003797 37438092

[16] Webb BJ, Sorensen J, Jephson A, Mecham I, Dean NC. Broad-spectrum antibiotic use and poor outcomes in community-onset pneumonia: a cohort study. Eur Respir J. 2019;54(1):1900057. doi: 10.1183/13993003.00057-2019 31023851

[17] Takazono T, Hosogaya N, Saito Y, Takemura M, Iwanaga N, Sakamoto N, et al. Effects of broad-spectrum antimicrobials on patients with community-acquired pneumonia with low risk for drug-resistant pathogens: historical cohort study in Japan. Infect Dis Ther. 2025;14(5):1043–59. doi: 10.1007/s40121-025-01142-1 40183917 PMC12084438

[18] Baghdadi JD, Goodman KE, Magder LS, Claeys KC, Sutherland ME, Harris AD. Association between delayed broad-spectrum gram-negative antibiotics and clinical outcomes: how much does getting it right with empiric antibiotics matter?. Clin Infect Dis. 2025;80(5):949–58. doi: 10.1093/cid/ciaf039 39874272 PMC12135916

[19] Aoto K, Inose R, Kosaka T, Shikata K, Muraki Y. Comparative effectiveness of cefmetazole versus carbapenems and piperacillin/tazobactam as initial therapy for bacteremic acute cholangitis: A retrospective study. J Infect Chemother. 2024;30(3):213–8. doi: 10.1016/j.jiac.2023.10.007 37832824

[20] Schneeweiss S, Avorn J. A review of uses of health care utilization databases for epidemiologic research on therapeutics. J Clin Epidemiol. 2005;58(4):323–37. doi: 10.1016/j.jclinepi.2004.10.012 15862718

[21] Mizuno K, Inose R, Goto R, Muraki Y. Adherence to guidelines for antibiotics used in the initial treatment of febrile neutropenia in patients with cancer: a study using health insurance claims database in Japan. J Pharm Health Care Sci. 2025;11(1):47. doi: 10.1186/s40780-025-00455-0 40481604 PMC12144810

[22] JMDC Real World. Hospital database. Accessed 2025 October 23. https://www.eng.phm-jmdc.com/hospital-database

[23] Langan SM, Schmidt SA, Wing K, Ehrenstein V, Nicholls SG, Filion KB, et al. The reporting of studies conducted using observational routinely collected health data statement for pharmacoepidemiology (RECORD-PE). BMJ. 2018;363:k3532. doi: 10.1136/bmj.k3532 30429167 PMC6234471

[24] Tamma PD, Avdic E, Li DX, Dzintars K, Cosgrove SE. Association of adverse events with antibiotic use in hospitalized patients. JAMA Intern Med. 2017;177(9):1308–15. doi: 10.1001/jamainternmed.2017.1938 28604925 PMC5710569

[25] Quan H, Sundararajan V, Halfon P, Fong A, Burnand B, Luthi J-C, et al. Coding algorithms for defining comorbidities in ICD-9-CM and ICD-10 administrative data. Med Care. 2005;43(11):1130–9. doi: 10.1097/01.mlr.0000182534.19832.83 16224307

[26] Takahashi N, Imaeda T, Oami T, Abe T, Shime N, Komiya K, et al. Incidence and mortality of community-acquired and nosocomial infections in Japan: a nationwide medical claims database study. BMC Infect Dis. 2024;24(1):518. doi: 10.1186/s12879-024-09353-6 38783190 PMC11112762

[27] Charlson ME, Pompei P, Ales KL, MacKenzie CR. A new method of classifying prognostic comorbidity in longitudinal studies: development and validation. J Chronic Dis. 1987;40(5):373–83. doi: 10.1016/0021-9681(87)90171-8 3558716

[28] Tagashira Y, Sakamoto N, Isogai T, Hikone M, Kosaka A, Chino R, et al. Impact of inadequate initial antimicrobial therapy on mortality in patients with bacteraemic cholangitis: a retrospective cohort study. Clin Microbiol Infect. 2017;23(10):740–7. doi: 10.1016/j.cmi.2017.02.027 28254686

[29] O’Brien RM. A caution regarding rules of thumb for variance inflation factors. Qual Quant. 2007;41:673–90. doi: 10.1007/s11135-006-9018-6

[30] D’Agostino RB Jr. Propensity score methods for bias reduction in the comparison of a treatment to a non-randomized control group. Stat Med. 1998;17(19):2265–81. doi: 10.1002/(sici)1097-0258(19981015)17:19<2265::aid-sim918>3.0.co;2-b 9802183

[31] Sader HS, Flamm RK, Carvalhaes CG, Castanheira M. Antimicrobial susceptibility of pseudomonas aeruginosa to ceftazidime-avibactam, ceftolozane-tazobactam, piperacillin-tazobactam, and meropenem stratified by U.S. census divisions: results from the 2017 INFORM program. Antimicrob Agents Chemother. 2018;62(12):e01587-18. doi: 10.1128/AAC.01587-18 30224535 PMC6256773

[32] Ministry of Health, Labour and Welfare. Japan nosocomial infections surveillance, JANIS open report. Accessed 2026 January 22. https://janis.mhlw.go.jp/english/report/index.html

[33] Harris PNA, Tambyah PA, Lye DC, Mo Y, Lee TH, Yilmaz M, et al. Effect of piperacillin-tazobactam vs meropenem on 30-day mortality for patients with E coli or klebsiella pneumoniae bloodstream infection and ceftriaxone resistance: a randomized clinical trial. JAMA. 2018;320(10):984–94. doi: 10.1001/jama.2018.12163 30208454 PMC6143100

[34] Hamilton WL, Pires S-M, Lippett S, Gudka V, Cross ELA, Llewelyn MJ. The impact of diagnostic microbiology on de-escalation of antimicrobial therapy in hospitalised adults. BMC Infect Dis. 2020;20(1):102. doi: 10.1186/s12879-020-4823-4 32013908 PMC6998081

[35] Krockow EM, Colman AM, Chattoe-Brown E, Jenkins DR, Perera N, Mehtar S, et al. Balancing the risks to individual and society: a systematic review and synthesis of qualitative research on antibiotic prescribing behaviour in hospitals. J Hosp Infect. 2019;101(4):428–39. doi: 10.1016/j.jhin.2018.08.007 30099092

[36] Salsgiver E, Bernstein D, Simon MS, Eiras DP, Greendyke W, Kubin CJ, et al. Knowledge, attitudes, and practices regarding antimicrobial use and stewardship among prescribers at acute-care hospitals. Infect Control Hosp Epidemiol. 2018;39(3):316–22. doi: 10.1017/ice.2017.317 29402339

[37] Takamatsu A, Yao K, Murakami S, Tagashira Y, Hasegawa S, Honda H. Barriers to adherence to antimicrobial stewardship postprescription review and feedback for broad-spectrum antimicrobial agents: a nested case-control study. Open Forum Infect Diseases. 2020;7(8). doi: 10.1093/ofid/ofaa298 PMC7434090 32832576

[38] Sutton JD, Lee JH, Howard K, Quartuccio KS, McCoy C, Gupta A, et al. Variability in durations of therapy for gram-negative bloodstream infections and factors associated with prolonged durations. Open Forum Infect Dis. 2025;12(6):ofaf298. doi: 10.1093/ofid/ofaf298 40476029 PMC12138504

