# Supplementary material for: Clinical impact of broad- versus narrow-spectrum empiric therapy in acute cholangitis: A Japanese claims database study
Source: PLoS One. 2026 Apr 2;21(4):e0346452. doi: 10.1371/journal.pone.0346452 (PMC13046160; doi:10.1371/journal.pone.0346452)
Supplement: S1 Table — (DOCX) [file pone.0346452.s002.docx]

**S1 Table. Antibiotics included in the analysis**

| Group | Classification | Antibiotics | Anatomical therapeutic chemical code |
| --- | --- | --- | --- |
| Narrow-spectrum group | Combinations of penicillins and beta-lactamase inhibitors | Ampicillin/sulbactam | J01CR01 |
|  | First-generation cephalosporins | Cefazolin | J01DB04 |
|  | Second-generation cephalosporins | Cefotiam | J01DC07 |
|  |  | Cefmetazole | J01DC09 |
|  |  | Flomoxef | J01DC14 |
|  | Third-generation cephalosporins | Cefotaxime | J01DD01 |
|  |  | Ceftazidime | J01DD02 |
|  |  | Ceftriaxone | J01DD04 |
|  |  | Cefoperazone/sulbactam | J01DD62 |
|  | Fluoroquinolones | Ciprofloxacin | J01MA02 |
|  |  | Levofloxacin | J01MA12 |
|  |  | Pazufloxacin | J01MA18 |
|  | Monobactams | Aztreonam | J01DF01 |
| Broad-spectrum group | Fourth-generation cephalosporins | Cefepime | J01DE01 |
|  |  | Cefozopran | J01DE03 |
|  | Combinations of penicillins and beta-lactamase inhibitors | Piperacillin/tazobactam | J01CR05 |
|  | Carbapenems | Meropenem | J01DH02 |
|  |  | Doripenem | J01DH04 |
|  |  | Imipenem/cilastatin | J01DH51 |
