# Supplementary material for: Clinical impact of broad- versus narrow-spectrum empiric therapy in acute cholangitis: A Japanese claims database study
Source: PLoS One. 2026 Apr 2;21(4):e0346452. doi: 10.1371/journal.pone.0346452 (PMC13046160; doi:10.1371/journal.pone.0346452)
Supplement: S2 Table — (DOCX) [file pone.0346452.s003.docx]

**S2 Table. Codes used for variable definitions**

| Variables | Definition |
| --- | --- |
| Acute cholangitis^a^ | K803, K830 |
| Blood culture^b^ | 160058610 |
| Biliary decompression^b^ | 150174550, 150362310, 150254410, 150437870 |
| Sepsis^a^ | A41, D65, I46, J80, J96, K72, N00, N01, N17, R40, R55, R57, D695, E872, G934, I951, J952, J953, R092, R418 |
| Vasopressors^c^ | C01CA01, C01CA02, C01CA03, C01CA04,  C01CA06, C01CA07, C01CA24, H01BA01 |
| ICU admission^a^ | A301, A301-2 |
| Immunosuppressants^c^ | H02, L01, L04 |
| Antibiotics^c^ | J01 (Vancomycin; J01XA01) |
| CDI^a^ | A047 |

Abbreviations: CDI, *Clostridioides difficile* infection; ICU, intensive care unit.

^a^ International Classification of Diseases, 10th Edition code. ^b^ Japan-specific standardized procedure code. ^c^ Anatomical therapeutic chemical code.
