# Supplementary material for: Clinical impact of broad- versus narrow-spectrum empiric therapy in acute cholangitis: A Japanese claims database study
Source: PLoS One. 2026 Apr 2;21(4):e0346452. doi: 10.1371/journal.pone.0346452 (PMC13046160; doi:10.1371/journal.pone.0346452)
Supplement: S1 File — (DOCX) [file pone.0346452.s004.docx]

**S1** **Supporting Information –A sensitivity analysis in the patient selection process –**

We examined the baseline characteristics and 30-day in-hospital mortality rates of patients who did not undergo blood culture collection. Of the 23,609 patients excluded due to a lack of blood culture collection, 17,177 and 73 patients were further excluded following the same criteria used in the primary analysis: failure to undergo biliary drainage within 2 days after the initiation of antimicrobial therapy and unknown discharge dates, respectively (Fig A). Consequently, 6,359 patients (5,509 and 850 in the narrow- and broad-spectrum groups, respectively) were included in the analysis.

**
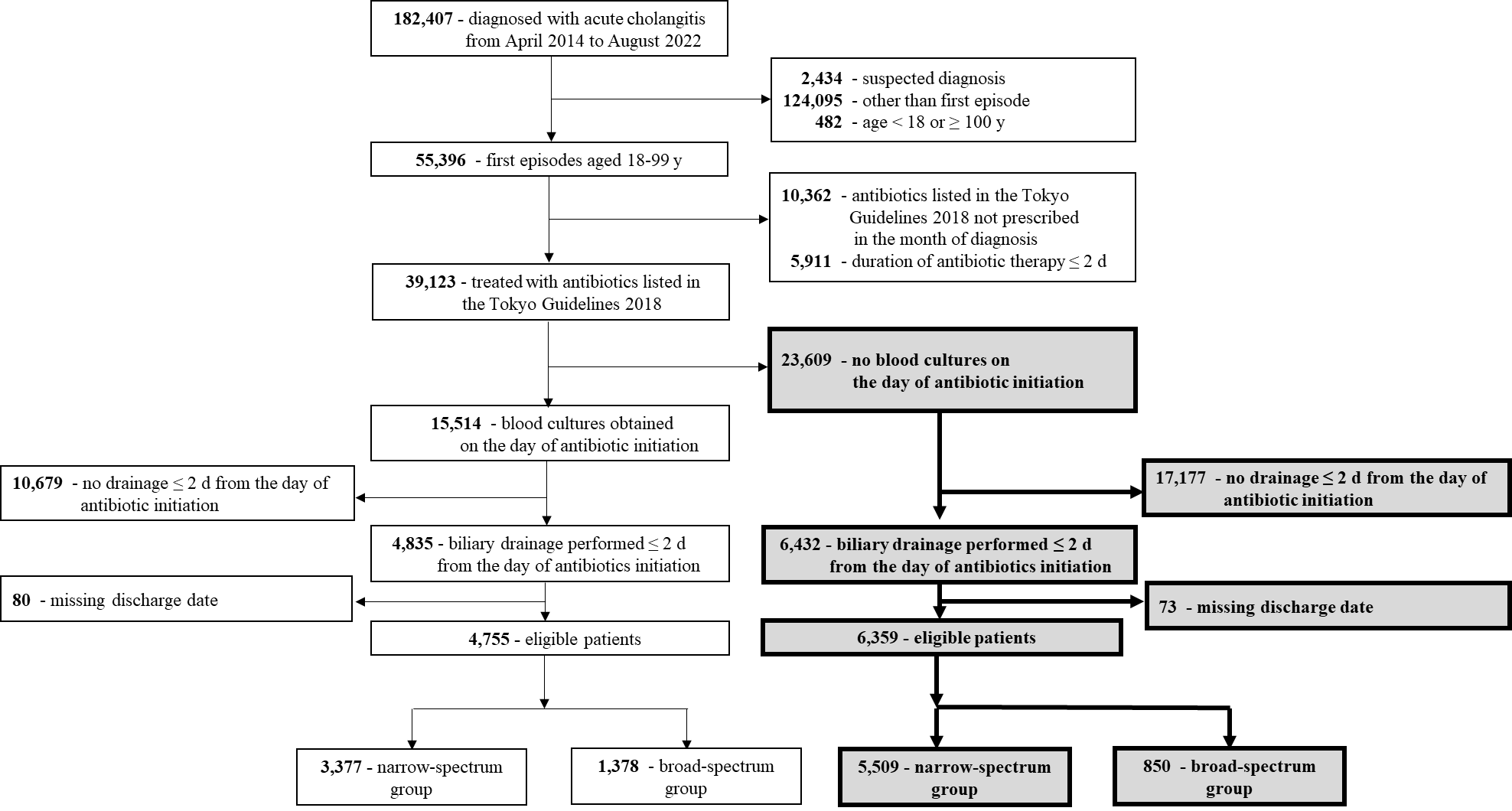
**

**Fig A. Patient selection process in a sensitivity analysis.**

The patient selection process in the sensitivity analysis was shown on a gray background.

Table A shows baseline characteristics of patients in this analysis. Compared to the primary analysis, the proportion of patients in the broad-spectrum group was relatively lower than that in the narrow-spectrum group. In addition, the proportions of patients aged ≥75, those with sepsis, and those admitted to the ICU were relatively low, and no patients received vasopressors (Table A). The 30-day in-hospital mortality rates were 0.82% (n = 45) and 2.2% (n = 19) in the narrow- and broad-spectrum groups, respectively, and were not higher than those in the results of primary analysis. These findings suggest that this cohort may include a large number of mild cases. Therefore, it is considered that the reason blood cultures were not collected was likely not due to severe illness, but rather because the illness was mild. Consequently, the patient selection process in this study is considered unlikely to introduce major selection bias.

**Table A. Baseline characteristics of the patients in a sensitivity analysis.**

|  | **Narrow-spectrum group**  **(n = 5509)** | **Broad-spectrum group**  **(n = 850)** |
| --- | --- | --- |
| **Demographics** |  |  |
| **Male sex** | 2951 (53.6) | 479 (56.4) |
| **Age, y, median (IQR)** | 81 (71-87) | 82 (73-88) |
| **Age ≥ 75 y** | 3660 (66.4) | 611 (71.9) |
| **Community-acquired cholangitis** | 5308 (96.4) | 822 (96.7) |
| **CCI, median (IQR)** | 1 (0-2) | 1 (0-2) |
| **Sepsis** | 527 (9.6) | 198 (23.3) |
| **Vasopressor prescription** | 0 (0) | 0 (0) |
| **ICU admission** | 86 (1.6) | 45 (5.3) |
| **History of prescriptions** |  |  |
| Immunosuppressant(s) | 445 (8.1) | 79 (9.3) |
| Antibiotic(s) | 79 (9.3) | 524 (8.2) |
| **Combination antibiotics** |  |  |
| Vancomycin | 0 (0) | 0 (0) |
| **Hospital bed count** |  |  |
| ≤ 199 | 729 (13.2) | 91 (10.7) |
| 200–499 | 3381 (61.4) | 469 (55.2) |
| ≥ 500 | 1398 (25.4) | 290 (34.1) |
| **Treatment year** |  |  |
| 2014 | 208 (3.8) | 24 (2.8) |
| 2015 | 282 (5.1) | 33 (3.9) |
| 2016 | 342 (6.2) | 40 (4.7) |
| 2017 | 483 (8.8) | 51 (6.0) |
| 2018 | 873 (15.8) | 126 (14.8) |
| 2019 | 963 (17.5) | 156 (18.4) |
| 2020 | 927 (16.8) | 181 (21.3) |
| 2021 | 954 (17.3) | 142 (16.7) |
| 2022 | 208 (3.8) | 24 (2.8) |

Data are presented as numbers (%) unless otherwise indicated.

Abbreviations: CCI, Charlson Comorbidity Index; ICU, intensive care unit; IQR, interquartile range.
