# Supplementary material for: Clinical impact of broad- versus narrow-spectrum empiric therapy in acute cholangitis: A Japanese claims database study
Source: PLoS One. 2026 Apr 2;21(4):e0346452. doi: 10.1371/journal.pone.0346452 (PMC13046160; doi:10.1371/journal.pone.0346452)
Supplement: S3 Table — (DOCX) [file pone.0346452.s005.docx]

**S3 Table. Baseline characteristics of the patients before and after PS matching**

|  | Before PS matching | | | After PS matching | | |
| --- | --- | --- | --- | --- | --- | --- |
|  | Narrow-spectrum group  (n = 3377) | Broad-spectrum group  (n = 1378) | ASD | Narrow-spectrum group  (n = 1378) | Broad-spectrum group  (n = 1378) | ASD |
| Demographics |  |  |  |  |  |  |
| Male sex | 1864 (55.2) | 753 (54.6) | 0.011 | 727 (52.8) | 753 (54.6) | 0.038 |
| Age, y, median (IQR) | 81 (73–87) | 82 (75–88) | 0.144 | 83 (76–88) | 82 (75–88) | 0.039 |
| Age ≥ 75 y | 2418 (71.6) | 1058 (76.8) | 0.118 | 1082 (78.5) | 1058 (76.8) | 0.040 |
| Community-acquired cholangitis | 3187 (94.4) | 1254 (91.0) | 0.130 | 1253 (90.9) | 1254 (91.0) | 0.003 |
| CCI, median (IQR) | 1 (0–2) | 1 (0–2) | 0.008 | 1 (0–2) | 1 (0–2) | 0.008 |
| Sepsis | 743 (22.0) | 489 (35.5) | 0.301 | 478 (34.7) | 489 (35.5) | 0.018 |
| Vasopressor prescription | 162 (4.8) | 87 (6.3) | 0.066 | 78 (5.7) | 87 (6.3) | 0.029 |
| ICU admission | 106 (3.1) | 106 (7.7) | 0.202 | 82 (6.0) | 106 (7.7) | 0.077 |
| History of prescriptions |  |  |  |  |  |  |
| Immunosuppressant(s) | 259 (7.7) | 161 (11.7) | 0.136 | 131 (9.5) | 161 (11.7) | 0.074 |
| Antibiotic(s) | 401 (11.9) | 201 (14.6) | 0.080 | 186 (13.5) | 201 (14.6) | 0.032 |
| Combination antibiotics |  |  |  |  |  |  |
| Vancomycin | 6 (0.2) | 6 (0.4) | 0.047 | 4 (0.3) | 6 (0.4) | 0.026 |
| Hospital bed count |  |  |  |  |  |  |
| ≤ 199 | 221 (6.5) | 109 (7.9) | 0.053 | 102 (7.4) | 109 (7.9) | 0.020 |
| 200–499 | 1957 (58.0) | 668 (48.5) | 0.191 | 676 (49.1) | 668 (48.5) | 0.012 |
| ≥ 500 | 1199 (35.5) | 601 (43.6) | 0.166 | 600 (43.5) | 601 (43.6) | 0.001 |
| Treatment year |  |  |  |  |  |  |
| 2014 | 80 (2.4) | 23 (1.7) | 0.050 | 26 (1.9) | 23 (1.7) | 0.015 |
| 2015 | 138 (4.1) | 34 (2.5) | 0.091 | 34 (2.5) | 34 (2.5) | 0.000 |
| 2016 | 167 (4.9) | 57 (4.1) | 0.039 | 50 (3.6) | 57 (4.1) | 0.024 |
| 2017 | 220 (6.5) | 70 (5.1) | 0.061 | 63 (4.6) | 70 (5.1) | 0.022 |
| 2018 | 499 (14.8) | 218 (15.8) | 0.029 | 209 (15.2) | 218 (15.8) | 0.018 |
| 2019 | 590 (17.5) | 211 (15.3) | 0.058 | 223 (16.2) | 211 (15.3) | 0.024 |
| 2020 | 656 (19.4) | 267 (19.4) | 0.001 | 276 (20.0) | 267 (19.4) | 0.017 |
| 2021 | 699 (20.7) | 317 (23.0) | 0.056 | 302 (21.9) | 317 (23.0) | 0.026 |
| 2022 | 328 (9.7) | 181 (13.1) | 0.108 | 195 (14.2) | 181 (13.1) | 0.032 |

Data are presented as numbers (%) unless otherwise indicated.

The PS was estimated by logistic regression using the following variables: age, sex, acquisition type, CCI, presence of sepsis, vasopressor prescription, ICU admission, history of immunosuppressant or antibiotic prescription within 90 days, hospital bed count, and treatment year.

Abbreviations: ASD, absolute standardized difference; CCI, Charlson Comorbidity Index; ICU, intensive care unit; IQR, interquartile range; PS, propensity score.
