# Supplementary material for: Clinical impact of broad- versus narrow-spectrum empiric therapy in acute cholangitis: A Japanese claims database study
Source: PLoS One. 2026 Apr 2;21(4):e0346452. doi: 10.1371/journal.pone.0346452 (PMC13046160; doi:10.1371/journal.pone.0346452)
Supplement: S4 Table — (DOCX) [file pone.0346452.s006.docx]

**S4 Table. Antibiotics included in the narrow-spectrum and broad-spectrum groups after PS matching**

| Group | Classification | Antibiotics | n (%) |
| --- | --- | --- | --- |
| **Narrow-spectrum group** |  |  | **1378 (100)** |
|  | Combinations of penicillins and beta-lactamase inhibitors | Ampicillin/sulbactam | 183 (13.28) |
|  | First-generation cephalosporins | Cefazolin | 5 (0.36) |
|  | Second-generation cephalosporins | Cefmetazole | 285 (20.68) |
|  |  | Cefotiam | 13 (0.94) |
|  |  | Flomoxef | 1 (0.07) |
|  | Third-generation cephalosporins | Cefoperazone/sulbactam | 819 (59.43) |
|  |  | Ceftriaxone | 55 (3.99) |
|  |  | Cefotaxime | 5 (0.36) |
|  |  | Ceftazidime | 3 (0.22) |
|  | Fluoroquinolones | Ciprofloxacin | － |
|  |  | Levofloxacin | 8 (0.58) |
|  | Monobactams | Aztreonam | 1 (0.07) |
| **Broad-spectrum group** |  |  | **1378 (100)** |
|  | Fourth-generation cephalosporins | Cefepime | 14 (1.02) |
|  |  | Cefozopran | 13 (0.94) |
|  | Combinations of penicillins and beta-lactamase inhibitors | Piperacillin/tazobactam | 813 (59.00) |
|  | Carbapenems | Meropenem | 465 (33.74) |
|  |  | Doripenem | 62 (4.50) |
|  |  | Imipenem/cilastatin | 11 (0.80) |
