# Supplementary material for: Clinical impact of broad- versus narrow-spectrum empiric therapy in acute cholangitis: A Japanese claims database study
Source: PLoS One. 2026 Apr 2;21(4):e0346452. doi: 10.1371/journal.pone.0346452 (PMC13046160; doi:10.1371/journal.pone.0346452)
Supplement: S1 Fig — (PDF) [file pone.0346452.s007.pdf]

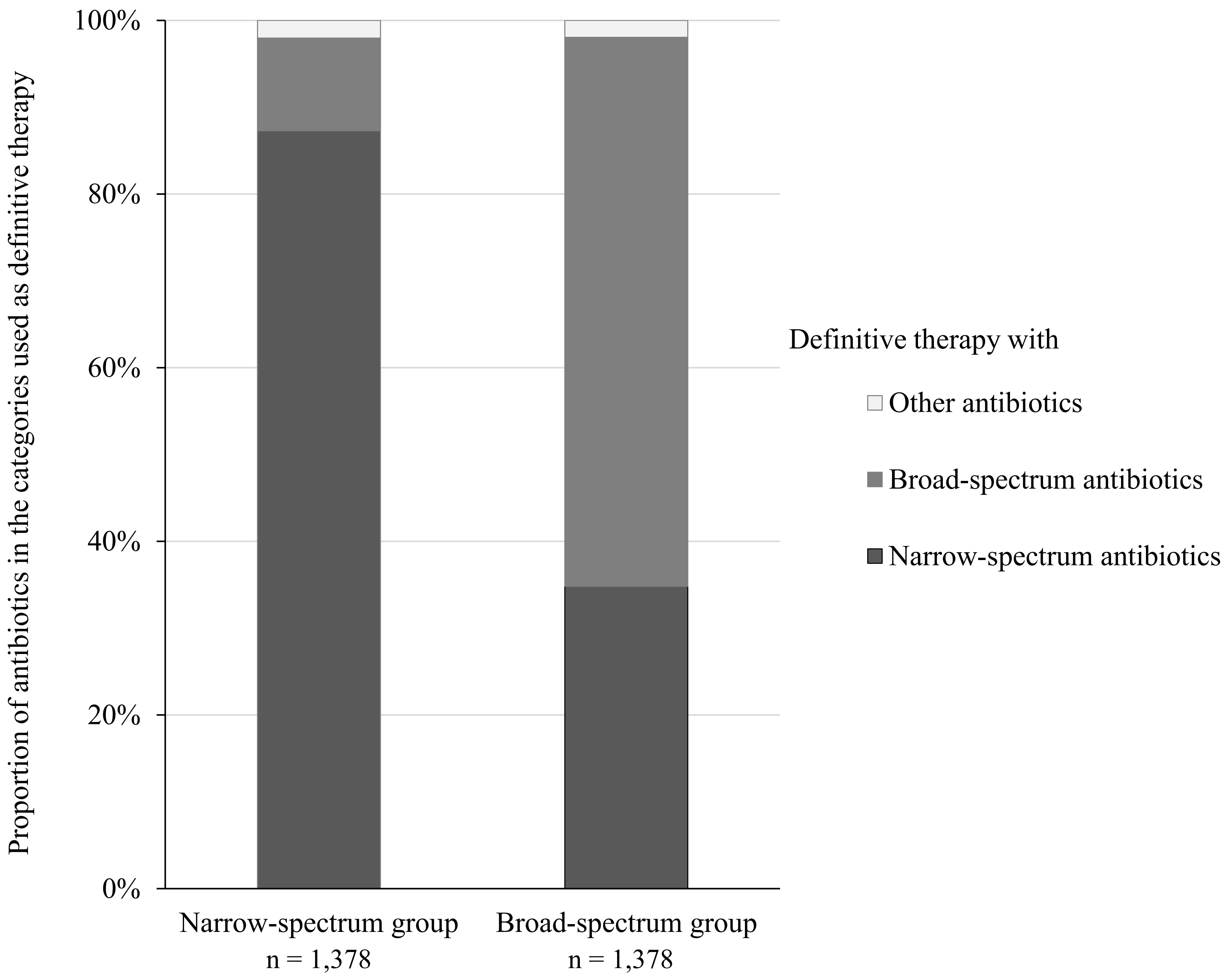

S1 Fig. Proportions of antibiotics in the categories used as definitive therapy stratified by empiric therapy
