# Supplementary material for: Clinical impact of broad- versus narrow-spectrum empiric therapy in acute cholangitis: A Japanese claims database study
Source: PLoS One. 2026 Apr 2;21(4):e0346452. doi: 10.1371/journal.pone.0346452 (PMC13046160; doi:10.1371/journal.pone.0346452)
Supplement: Attachment — Submitted filename: Review_Plosone.docx [file pone.0346452.s008.docx]

**Review:**

This study addresses an important and clinically relevant question regarding empiric antibiotic selection in patients with acute cholangitis. The use of a large, nationwide Japanese claims database provides a robust sample size and enhances the generalizability of the findings within the studied healthcare setting. The authors employ appropriate and well-established statistical methods, including multivariable logistic regression and propensity score matching, to account for confounding in this observational design. The inclusion of clinically meaningful outcomes, such as 30-day in-hospital mortality and healthcare utilization measures, further strengthens the study. However, the manuscript would benefit from a more comprehensive review of the existing literature and greater clarity in the definition and explanation of key exposure and outcome measures, which would improve contextualization of the findings and overall interpretability. Overall, the study contributes valuable evidence to ongoing discussions around antimicrobial stewardship in the management of acute cholangitis.

**Below are some specific comments and recommendations:**

Introduction: The introduction would benefit from additional detail and stronger integration of the existing scientific literature. Specifically, the background section lacks sufficient evidence-based justification to clearly establish the scientific context, knowledge gaps, and rationale for the investigation being reported. Incorporating more up-to-date and relevant references, along with a clearer articulation of how this study addresses unmet needs or limitations in prior research, would substantially strengthen the motivation and framing of the study.

Line 55: Add citation

Line 59-60: Rephrase for clarity. What you mean by appropriateness? Does it refer to incorrect therapy selection for a pathogen of interest that increases mortality chances?

Line 63: Add citation

Line 63-68: These seem to appear as two objective statements that partially overlap but are phrased differently. This is confusing. Consider consolidating these into a single, coherent objective or clearly define one as an overarching aim and the other as a specific objective, if that is the case. Also consider adding the evaluation of secondary outcomes as secondary objectives.

Line 77: “We accessed the database from January 23, 2025”. Sentence is misleading. Consider reframing to something like The database was accessed on January 23, 2025, for data extraction.

Line 86: change diagnosis to diagnosed

Line 91: consider adding context to why discharge date was included as an eligibility criterion for clarity

Line 98: Primary outcome is 30-day in-hospital mortality rate or 30-day in-hospital mortality. Be consistent. Given the analysis, it seems like a binary variable rather than a rate. Please clarify.

Line 98-99: The secondary outcomes included the incidence of Clostridioides difficile infection (CDI) during hospitalization. There is no context in the introduction or methods as to why this is being evaluated. Consider adding some context.

Line 101: consider defining empiric and definitive therapy in brief

Line 108: define CCI and provide context

Line 112: Is the 2 days period and empiric therapy initiation based on culture results? If so, please explicitly mention that.

Line 105 and line 118 suggest that empiric and definitive therapy is determined based on when the antibiotic is given rather than which one and on what basis? I am assuming this too is influenced based on culture results.

Line 123-124: clearly specify your outcome and exposure variables and consider adding categories in brackets. For instance, 30 day hospital mortality (yes/no).

Line 128-130: add citation for propensity score matching

Under statistical analysis, please clarify the method used for multivariable model building, the significance threshold applied in the final mode, and whether multicollinearity among covariates were assessed (like presence of sepsis and ICu admissions may be associated)

Line 150-152: add values and table reference. 3.6 % difference does not seem like a large proportion for ICU admissions.

Line 152: table 2 introductory line missing

Line 174 & 175: consider adding significantly associated and not significantly associated.

Table 3: Add significance level for p-value in footnotes, specify unit for age in the table,

Line 187: consider adding not significantly associated.

Line 189: “Table 4 presents the results regarding secondary outcomes” sentence is incomplete/unclear. Results of which test? Same for table caption

Table 4: Add significance level for p-value in footnotes,

Line 213: Was not associated with **increased** 30-day in hospital mortality

Line 216: reference results table

Line 221: add citation

Line 223: reference results table

Line 230: 10 % may not be considered rare. Consider changing rare to lower or uncommon. Also add reference results table.

Line 232: As the study results do not directly demonstrate low resistance, consider revising the wording to reflect a lower likelihood of resistant pathogens or a higher likelihood of pathogen susceptibility.

Line 234- 238: The discussion suggests that broad-spectrum empiric therapy leads to unnecessary continuation of antibiotics based on negative blood cultures; however, this conclusion appears speculative and does not account for other factors influencing de-escalation decisions like limited microbiological data or CCI, sepsis, or ICU admissions.

Line 246: reference results table

Line 252: add citation

Line 260: Add Implication of this limitation on study results

The repeated characterization of broad-spectrum antibiotic use as “unnecessary” in the Discussion and Conclusion may overstate the findings. Consider revising the wording to use more neutral terms that better reflect the observational nature of the study (suggestions: avoid prolonged use or cautious/ culture guided use is warranted).
