# Supplementary material for: Clinical impact of broad- versus narrow-spectrum empiric therapy in acute cholangitis: A Japanese claims database study
Source: PLoS One. 2026 Apr 2;21(4):e0346452. doi: 10.1371/journal.pone.0346452 (PMC13046160; doi:10.1371/journal.pone.0346452)
Supplement: Attachment — Submitted filename: Response to Reviewers_aoto_260216 1.docx [file pone.0346452.s010.docx]

**Response to Reviewer 1**

Thank you for taking the time to review our manuscript. We appreciate your helpful suggestions regarding various issues, including various biases and descriptions of the results. In response to your comments, we have revised the contents of the manuscript. Our revisions and comments are as follows. The changes in the revised manuscript are highlighted. We would appreciate your confirmation.

***Comment 1***

*1. Justification of exclusions (selection bias)*

*The patient selection process excluded 23,609 patients because they did not have blood cultures collected on the day of antibiotic initiation. This represents a significant portion of the original population. From the reviewer’s knowledge, it is crucial to determine if clinicians systematically omit blood cultures in less severe cases or, conversely, in cases so critical that immediate intervention precludes culture collection.*

*- Please provide a sensitivity analysis comparing the baseline characteristics and 30-day mortality of the 23,609 excluded patients with the 4,755 included patients.*

**Response 1:**

Thank you for your comments. The database used in this study contained only monthly diagnostic data and did not include detailed daily diagnostic information. Therefore, to enhance the reliability of the diagnosis of acute cholangitis, we included patients who had blood cultures collected on the day antimicrobial therapy was initiated, and further restricted the analysis to those who underwent biliary drainage within 2 days after the initiation of antimicrobial therapy. These approaches are recommended in clinical guidelines for the management of moderate-to-severe cholangitis (PMID: 28941329). Therefore, we believe that this patient selection process more accurately reflects real-world clinical practice in the treatment of acute cholangitis.

Following your helpful comments, we have conducted a sensitivity analysis. The results of this sensitivity analysis are shown in S1 Supporting Information. Of the 23,609 patients excluded due to a lack of blood culture collection, 17,177 and 73 patients were further excluded following the same criteria used in the primary analysis: failure to undergo biliary drainage within 2 days after the initiation of antimicrobial therapy and unknown discharge dates, respectively (Fig A in S1 Supporting Information). Consequently, 6,359 patients (5,509 and 850 in the narrow- and broad-spectrum groups, respectively) were included in the analysis. Table A in S1 Supporting Information shows the baseline characteristics of the patients included in this analysis. Compared to the primary analysis, the proportion of patients in the broad-spectrum group was relatively lower than that in the narrow-spectrum group. Furthermore, the proportions of patients aged ≥75 years, those with sepsis, and those admitted to the ICU were relatively low, and no patients received vasopressors (Table A in S1 Supporting Information). The 30-day in-hospital mortality rates were 0.82% (n = 45) and 2.2% (n = 19) in the narrow- and broad-spectrum groups, respectively, which were not higher than those in the results of primary analyses. These findings suggested that this cohort may have included a large number of mild cases. Therefore, it is considered that the reason blood cultures were not collected was likely not due to severe illness, but rather because the illness was mild. Consequently, the patient selection process in this study was unlikely to introduce a major selection bias.

Incidentally, many patients who did not undergo biliary drainage were excluded from this sensitivity analysis. These patients were outside the scope of this study because it was unclear whether they had received antibiotics to treat acute cholangitis.

To explain the patient selection process and this sensitivity analysis, we have revised the Materials and Methods section and the Results section and added S1 Supporting Information. In accordance with this revision, we have moved the description of the 30-day in-hospital mortality rate and revised the wording to improve readability.

**Before:** Materials and Methods (Lines 87-90)

The inclusion criteria were as follows: age 18–99 years, first episode of acute cholangitis, receiving antibiotics listed in the Tokyo Guidelines 2018 for more than 2 days [10], blood culture obtained on the day of antibiotic initiation, and biliary drainage performed within 2 days of antibiotic initiation.

**After:** Materials and Methods (Lines 102-107)

The database used in this study contained only monthly diagnostic data and did not include detailed daily diagnostic information. Therefore, to enhance the reliability of the acute cholangitis diagnosis, the following inclusion criteria were adopted: age 18–99 years, first episode of acute cholangitis, receiving antibiotics listed in the Tokyo Guidelines 2018 for more than 2 days [10], blood culture obtained on the day of antibiotic initiation, and biliary drainage performed within 2 days of antibiotic initiation.

**Addition:** Materials and Methods (Lines 169-173)

Sensitivity analyses were performed to evaluate the robustness of the primary results. First, to rule out the possibility that blood cultures were not collected on the day of antimicrobial therapy initiation in severely ill patients, we examined the baseline characteristics and 30-day in-hospital mortality rates in patients without blood culture collection.

**Before:** Results (Lines 168-169)

The 30-day in-hospital mortality rates were 1.3% (n = 43) and 2.2% (n = 31) in the narrow-and broad-spectrum groups, respectively.

**After:** Results (Lines 197-200)

The 30-day in-hospital mortality rates in the narrow- and broad-spectrum groups were 1.3% (n = 43) and 2.2% (n = 31), respectively. S1 Supporting Information shows the results of a sensitivity analysis conducted in patients without blood culture collection.

**Addition:** S1 Supporting Information

Please refer to the S1 Supporting Information.

***Comment 2***

*2. Sensitivity analysis of the "sepsis" subgroup*

*The study correctly identifies that sepsis was significantly more prevalent in the broad-spectrum group (35.5% vs. 22.0%). While Propensity Score (PS) matching was used, the ‘benefit’ of broad-spectrum agents is often most pronounced in the highest-acuity patients. A subgroup-specific analysis would confirm if the "no benefit" finding holds true for the most vulnerable patients.*

*- Perform a sensitivity analysis restricted only to patients diagnosed with sepsis at the index date.*

**Response 2:**

As suggested, we have conducted a sensitivity analysis restricted to patients with sepsis to assess the robustness of the association between broad-spectrum empiric therapy and 30-day in-hospital mortality. Consistent with the primary analysis results, this result indicated that broad-spectrum empiric therapy was not associated with improved 30-day in-hospital mortality in patients with sepsis (OR, 1.48 [95% CI, 0.71–3.12], p = 0.30).

We have added the following description regarding this sensitivity analysis and have also revised the Discussion section.

**Addition:** Materials and Methods (Lines 173-175)

Second, a sensitivity analysis restricted to patients with sepsis in the PS-matched cohort was conducted to assess the robustness of the association between broad-spectrum empiric therapy and 30-day in-hospital mortality.

**Addition:** Results (Lines 225-227)

Furthermore, no significant association was observed even in a sensitivity analysis restricted to patients with sepsis (OR, 1.48 [95% CI, 0.71–3.12], p = 0.30).

**Before:** Discussion (Lines 213-214)

This finding was consistent across both the logistic regression and PS matching analyses, indicating the robustness of the results.

**After:** Discussion (Lines 255-258)

This finding was consistent across both the logistic regression and PS matching analyses. Furthermore, sensitivity analysis restricted to patients with sepsis showed similar results, supporting the robustness of the results.

***Comment 3***

*3. The length of hospital stay (LOS) and intravenous (IV) duration*

*The broad-spectrum group had significantly longer hospital stays (16 days vs. 13 days) and IV therapy duration (9 days vs. 8 days). Since 63.2% of the broad-spectrum group failed to de-escalate, the prolonged LOS may be tied more to stewardship failures than drug performance.*

*- Discuss whether this is a biological consequence of antibiotic efficacy or a behavioral consequence of clinician conservative bias when treating patients they perceive as high-risk.*

**Response 3:**

Thank you for your comments. We believe that the reason why many patients who received broad-spectrum empiric therapy failed to de-escalate is based on a behavioral consequence of clinician conservative bias, as previously reported (PMID: 30099092, PMID: 29402339).

To describe this point, we have revised the Discussion section.

**Before:** Discussion (Lines 235-240)

Considering that the positive blood culture rate for acute cholangitis is approximately 40% [1], broad-spectrum antibiotics may have been continued in patients with negative blood cultures. Thus, broad-spectrum empiric therapy may lead to unnecessary continuation of broad-spectrum antibiotics. Our findings suggest that the use of narrow-spectrum antibiotics, such as cefoperazone/sulbactam or cefmetazole, as empiric therapy for acute cholangitis can help reduce the use of piperacillin/tazobactam or meropenem.

**After:** Discussion (Lines 280-285)

Considering that the positive blood culture rate for acute cholangitis is approximately 40% [1], some patients in the broad-spectrum group may not have undergone de-escalation because their blood cultures were negative. Furthermore, the continuation of broad-spectrum antibiotics may be influenced by physicians’ strong sense of reassurance regarding the efficacy of these agents, as has been reported in studies on antibiotic prescription behavior [35,36].

***Comment 4***

*4. Causal interpretation, confounding, and generalizability*

*Given the observational design and limitations of claims data, readers could over‑interpret the findings as definitive evidence that broad‑spectrum empiric therapy is unnecessary in all cases of acute cholangitis. The manuscript would benefit from more precise framing of the target population and more explicit discussion of residual confounding and regional microbiology.*

*Please explicitly acknowledge:*

*- The likelihood of residual confounding by indication and severity, despite multivariable adjustment and PS matching, due to the absence of physiologic data, laboratory tests, and detailed organ dysfunction measures.*

*- That early and successful source control via drainage may diminish any incremental mortality benefit of broader empiric coverage, which limits generalizability to settings where drainage is delayed or less accessible.*

*- That the observed low prevalence of third‑generation cephalosporin resistance in E. coli and Klebsiella spp. and the low isolation rate of Pseudomonas in cholangitis in Japan may not hold in regions with higher resistance burdens, where empiric decisions may appropriately differ.*

**Response 4:**

As pointed out, this study has several limitations. We explicitly acknowledge these points and have revised the Discussion section accordingly.

Furthermore, regarding the third point raised, we have explicitly stated in the conclusion that the findings of this study are based on large-scale Japanese claims data. Accordingly, we have revised the abstract.

**Before:** Discussion (Lines 255-260)

First, residual confounders may have existed between the groups. To address the confounders, we performed multivariate logistic regression and PS matching analyses. However, disease severity may not have been adequately adjusted for because the database did not contain patients’ vital parameters and laboratory results. In addition, information regarding the diagnosis and treatment at institutions outside the database could not be collected and was unknown.

**After:** Discussion (Lines 304-310)

First, to address the confounders, we performed multivariate logistic regression and PS matching analyses. However, disease severity may not have been adequately adjusted for because the database did not contain patients’ vital parameters, laboratory results, and detailed organ dysfunction measures. In addition, information regarding the diagnosis and treatment at institutions outside the database could not be collected and was unknown. Therefore, residual confounding may exist based on the indications and severity.

**Before:** Discussion (Lines 262-265)

Consequently, our cohort may have included a higher proportion of patients with favorable prognoses. Therefore, our findings may not be generalizable to patients who do not undergo drainage. Moreover, it was unclear whether source control was successfully achieved after drainage.

**After:** Discussion (Lines 312-316)

Consequently, our cohort may have included a higher proportion of patients with favorable prognoses. Early and successful source control via drainage may offset the mortality-reducing effects of broad-spectrum empiric therapy, limiting its generalizability to situations in which drainage is delayed or unavailable. Additionally, it was unclear whether source control was successfully achieved after drainage.

**Before:** Discussion (Lines 250-254)

However, the clinical impact of broad-spectrum empiric therapy on patient outcomes may vary depending on regional resistance rates. Further research is warranted to evaluate the impact of broad-spectrum empiric therapy in regions with a high prevalence of antimicrobial resistance.

**After:** Discussion (Lines 318-321) (stated as a research limitation)

Third, our findings may have been influenced by the low prevalence of antimicrobial resistance in Japan. Broad-spectrum empiric therapy may be beneficial for patient outcomes in regions with a high prevalence of antimicrobial resistance. Further research is warranted to evaluate the impact of broad-spectrum empiric therapy in such settings.

**Before:** Conclusion (Lines 272-274)

This finding suggests that the necessity of broad-spectrum empiric therapy is limited and that narrow-spectrum empiric therapy may represent an effective treatment strategy for acute cholangitis.

**After:** Conclusion (Lines 327-330)

This finding, based on large-scale Japanese claims data, suggests that the necessity of broad-spectrum empiric therapy is limited and that narrow-spectrum empiric therapy may represent an effective treatment strategy for acute cholangitis.

**Before:** Abstract (Lines 40-42)

In this large-scale study using a claims database, broad-spectrum empiric therapy showed no clinical benefit, compared with narrow-spectrum empiric therapy.

**After:** Abstract (Lines 40-42)

In this large-scale study using a Japanese claims database, broad-spectrum empiric therapy was not associated with improved clinical outcomes, compared with narrow-spectrum empiric therapy.

***Comment 5***

*Lastly, please consider softening the definitive statements in the Conclusion of ‘broad-spectrum empiric therapy showed no clinical benefit’.*

**Response 5:**

As you suggested, we have revised the conclusion accordingly. Similarly, we have also softened the expressions in the Abstract and Discussion sections.

**Before:** Conclusion (Lines 271-272)

In this study, broad-spectrum empiric therapy showed no clinical benefit, compared with narrow-spectrum empiric therapy.

**After:** Conclusion (Lines 326-327)

In this study, broad-spectrum empiric therapy was not associated with improved clinical outcomes compared with narrow-spectrum empiric therapy.

**Before:** Abstract (Lines 40-42)

In this large-scale study using a claims database, broad-spectrum empiric therapy showed no clinical benefit, compared with narrow-spectrum empiric therapy.

**After:** Abstract (Lines 40-42)

In this large-scale study using a Japanese claims database, broad-spectrum empiric therapy was not associated with improved clinical outcomes, compared with narrow-spectrum empiric therapy.

**Before:** Discussion (Lines 216-217)

Therefore, no clinical benefit of broad-spectrum empiric therapy was demonstrated in this study.

**After:** Discussion (Lines 259-260)

Collectively, the present study did not identify any clear clinical benefits of broad-spectrum empiric therapy.

**Response to Reviewer 2:**

Thank you for taking the time to review our manuscript. We appreciate your helpful suggestions on many issues, including the various definitions, limitations, and descriptions of the results. In response to your comments, we have revised the contents of the manuscript. Our revisions and comments are as follows. The changes in the revised manuscript are highlighted. We would appreciate your confirmation.

***Comment 1***

*Introduction: The introduction would benefit from additional detail and stronger integration of the existing scientific literature. Specifically, the background section lacks sufficient evidence-based justification to clearly establish the scientific context, knowledge gaps, and rationale for the investigation being reported. Incorporating more up-to-date and relevant references, along with a clearer articulation of how this study addresses unmet needs or limitations in prior research, would substantially strengthen the motivation and framing of the study.*

**Response 1:**

Thank you for this helpful suggestion. Following your recommendations, we have added the latest and most relevant references to the Introduction and have substantially revised it to clarify the purpose of this study.

**Change:** Introduction (Lines 47-83)

Please refer to the Introduction section of the revised manuscript.

***Comment 2***

*Line 55: Add citation*

**Response 2:**

Thank you for your comments. Following the revisions to the Introduction, we have amended the relevant sentence as follows and added citations:

**Before:** Introduction (Lines 52-55)

To address the potential presence of antimicrobial resistance in these gram-negative pathogens, some clinicians select broad-spectrum antibiotics, such as carbapenems, piperacillin/tazobactam, or fourth-generation cephalosporins, for empiric therapy.

**After:** Introduction (Lines 53-56)

To address the potential presence of these antimicrobial resistance, clinical guidelines for acute cholangitis recommend empiric therapy with carbapenems, piperacillin/tazobactam, or fourth-generation cephalosporins [9,10].

***Comment 3***

*Line 59-60: Rephrase for clarity. What you mean by appropriateness? Does it refer to*

*incorrect therapy selection for a pathogen of interest that increases mortality chances?*

**Response 3:**

Thank you for your comments. As a result of the revisions to the Introduction section, the relevant sentence has been removed.

**Before:** Introduction (Lines 58-60)

In addition, some studies have reported that the appropriateness of empiric therapy for pathogens is associated with increased mortality [4,5], while others have shown no such association [6,7].

**After:** [Deletion]

***Comment 4***

*Line 63: Add citation*

**Response 4:**

Thank you for your comments. As a result of the revisions to the Introduction section, the relevant sentence has been removed.

**Before:** Introduction (Lines 62-63)

Assessing the necessity of broad-spectrum antibiotics is essential because their overuse contributes to the spread of antimicrobial resistance.

**After:** [Deletion]

***Comment 5***

*Line 63-68: These seem to appear as two objective statements that partially overlap but are phrased differently. This is confusing. Consider consolidating these into a single, coherent objective or clearly define one as an overarching aim and the other as a specific objective, if that is the case. Also consider adding the evaluation of secondary outcomes as secondary objectives.*

**Response 5:**

Based on your comments, we have revised the relevant sentences to clarify this study’s purpose. Additionally, we have added a description of the secondary objectives.

**Before:** Introduction (Lines 63-68)

Therefore, we used a Japanese claims database to investigate the association between administration of broad-spectrum empiric therapy and patient outcomes. To the best of our knowledge, no previous study has evaluated this association using multicenter data. The aim of this study was to compare the clinical impact of broad-spectrum empiric therapy with that of narrow-spectrum therapy in patients with acute cholangitis.

**After:** Introduction (Lines 79-83)

This study aimed to examine whether broad-spectrum empiric therapy is associated with improved 30-day in-hospital mortality compared with narrow-spectrum empiric therapy using a Japanese claims database. Furthermore, as a secondary objective, the impact of broad-spectrum empiric therapy on the incidence of CDI, duration of intravenous (IV) antibiotic therapy, and LOS was evaluated.

***Comment 6***

*Line 77: “We accessed the database from January 23, 2025”. Sentence is misleading. Consider reframing to something like The database was accessed on January 23, 2025, for data extraction.*

**Response 6:**

We have revised the relevant sentence according to your suggestion.

**Before:** Materials and Methods (Line 77)

We accessed the database from January 23, 2025.

**After:** Materials and Methods (Line 92)

Data extraction from the database began on January 23, 2025.

***Comment 7***

*Line 86: change diagnosis to diagnosed.*

**Response 7:**

We have revised the sentence according to your suggestion.

**Before:** Materials and Methods (Lines 86-87)

We enrolled patients diagnosis with acute cholangitis between April 2014 and August 2022.

**After:** Materials and Methods (Lines 101-102)

We enrolled patients diagnosed with acute cholangitis between April 2014 and August 2022.

***Comment 8***

*Line 91: consider adding context to why discharge date was included as an eligibility criterion for clarity*

**Response 8:**

As you suggested, we have added the reason for including discharge date in the eligibility criteria.

**Before:** Materials and Methods (Lines 90-91)

Patients with unknown discharge dates were excluded.

**After:** Materials and Methods (Lines 107-108)

Patients with unknown discharge dates were excluded as their outcomes could not be assessed.

***Comment 9***

*Line 98: Primary outcome is 30-day in-hospital mortality rate or 30-day in-hospital mortality. Be consistent. Given the analysis, it seems like a binary variable rather than a rate. Please clarify.*

**Response 9:**

As you have pointed out, the primary outcome was 30-day in-hospital mortality. We have revised this sentence as follows:

**Before:** Materials and Methods (Lines 97-98)

The primary outcome was the 30-day in-hospital mortality rate.

**After:** Materials and Methods (Lines 114-115)

The primary outcome was the 30-day in-hospital mortality.

***Comment 10***

*Line 98-99: The secondary outcomes included the incidence of Clostridioides difficile infection (CDI) during hospitalization. There is no context in the introduction or methods as to why this is being evaluated. Consider adding some context.*

**Response 10:**

Thank you for this helpful suggestion. We have added the description regarding CDI in the Introduction and Materials and Methods sections.

**Addition:** Introduction (Lines 61-63)

Such overuse not only contributes to the spread of antimicrobial resistance but may also be associated with higher mortality, an increased risk of *Clostridioides difficile* infection (CDI), and prolonged length of hospital stay (LOS) [11,14–17].

**Addition:** Introduction (Lines 81-83)

Furthermore, as a secondary objective, the impact of broad-spectrum empiric therapy on the incidence of CDI, duration of intravenous (IV) antibiotic therapy, and LOS was evaluated.

**Addition:** Materials and Methods (Lines 116-117)

CDI was evaluated as a representative adverse event associated with antimicrobial therapy [24].

***Comment 11***

*Line 101: consider defining empiric and definitive therapy in brief*

**Response 11:**

The definitions of empiric therapy and definitive therapy are described in the manuscript as follows: We would appreciate your confirmation.

**Lines 123-124:** antibiotic therapy initiated on the day of blood culture collection was considered empiric therapy,

**Lines 136-138:** Definitive therapy was defined as the antibiotic administered on the day of completion of IV antibiotic therapy.

***Comment 12***

*Line 108: define CCI and provide context*

**Response 12:**

We have added an explanation and citation regarding the Charlson Comorbidity Index in the Materials and Methods section.

**Addition:** Materials and Methods (Lines 124-129)

The following data were collected based on the index date: age; sex; acquisition type (community-acquired or healthcare-associated); Charlson Comorbidity Index (CCI) [25]; presence of sepsis; vasopressor prescription; intensive care unit (ICU) admission; concomitant prescription of vancomycin, history of immunosuppressant or antibiotic prescription within 90 days; hospital bed count; treatment year.

**Addition:** Materials and Methods (Lines 132-133)

The CCI was collected as a measure of comorbidity burden to assess the impact of comorbidities on prognosis [27].

***Comment 13***

*Line 112: Is the 2 days period and empiric therapy initiation based on culture results? If so, please explicitly mention that.*

**Response 13:**

Thank you for your comments. The 2-day period and initiation of empiric therapy are not based on culture results. The database used in this study did not contain information on culture results. Therefore, we have classified the acquisition type of acute cholangitis based on the interval between the date of admission and the initiation of empiric therapy. This classification method has been used in a previous study (PMID: 38783190). To clarify this point, we have added a citation. Additionally, we have clearly stated that no culture results were available in this study.

**Addition:** Materials and Methods (Lines 129-132)

Community-acquired cholangitis was defined as cases in which empiric therapy was initiated within 2 days of admission, whereas healthcare-associated cholangitis was defined as cases in which empiric therapy was initiated more than 2 days after admission [26].

**Before:** Materials and Methods (Lines 105-106)

Antibiotic therapy initiated on the day of blood culture collection was considered empiric therapy, and the date was defined as the index date.

**After:** Materials and Methods (Lines 122-124)

The database used in this study did not contain information on culture results. Therefore, antibiotic therapy initiated on the day of blood culture collection was considered empiric therapy, and the date was defined as the index date.

***Comment 14***

*Line 105 and line 118 suggest that empiric and definitive therapy is determined based on when the antibiotic is given rather than which one and on what basis? I am assuming this too is influenced based on culture results.*

**Response 14:**

Thank you for your comments. The database used in this study did not contain information on culture results. Consequently, empiric therapy and definitive therapy are defined based on the timing of administration. A similar definition has been used in a previous study (PMID: 39874272).

***Comment 15***

*Line 123-124: clearly specify your outcome and exposure variables and consider adding categories in brackets. For instance, 30 day hospital mortality (yes/no).*

**Response 15:**

As you suggested, we have revised the relevant section as follows.

**Addition:** Materials and Methods (Lines 144-145)

The exposure variable was the receipt of broad-spectrum empiric therapy (yes/no), and the outcome was 30-day in-hospital mortality (yes/no).

***Comment 16***

*Line 128-130: add citation for propensity score matching*

**Response 16:**

We have added a citation to the sentence.

**Addition:** Materials and Methods (Lines 151-153)

PS matching analysis was performed to adjust for the differences in baseline characteristics between the groups [30].

***Comment 17***

*Under statistical analysis, please clarify the method used for multivariable model building, the significance threshold applied in the final mode, and whether multicollinearity among covariates were assessed (like presence of sepsis and ICu admissions may be associated)*

**Response 17:**

Thank you for your comments. The method used for the multivariable model building was as follows: We would appreciate your confirmation.

**Lines 145-147:** Similar to a previous study [28], the multivariable model was adjusted for all variables with a p value ≤ 0.1 in the univariable analysis.

Although the significance threshold applied in the final model is also provided in the manuscript, we have revised the relevant description to clarify this level.

**Before:** Materials and Methods (Line 141)

Significance was defined as a two-sided p value < 0.05.

**After:** Materials and Methods (Line 164)

Significance in all statistical analyses was defined as a two-sided p value < 0.05.

Because multicollinearity assessment was not described, we have added this information to the Materials and Methods section. Additionally, in the Results section, we have stated that no concerning multicollinearity was identified based on this assessment.

**Addition:** Materials and Methods (Lines 147-149)

Multicollinearity among covariates was assessed by calculating the variance inflation factor for each variable, with values less than 10 considered to indicate no concerning multicollinearity [29].

**Addition:** Results (Lines 211-212)

Variance inflation factor values ranged from 1.01 to 1.17, indicating no concerning collinearity of the variables.

***Comment 18***

*Line 150-152: add values and table reference. 3.6 % difference does not seem like a large proportion for ICU admissions.*

**Response 18:**

Based on your comment, we have added these values and a reference to Table 1. We have also removed the description stating that a larger proportion of patients admitted to the ICU was found in the broad-spectrum group.

**Before:** Results (Lines 150-152)

A larger proportion of patients with sepsis and those admitted to the ICU were found in the broad-spectrum group than in the narrow-spectrum group.

**After:** Results (Lines 182-184)

A larger proportion of patients with sepsis was found in the broad-spectrum group (n =489 [35.5%]) than in the narrow-spectrum group (n =743 [22.0%]) (Table 1).

***Comment 19***

*Line 152: table 2 introductory line missing*

**Response 19:**

Thank you for pointing this out. We have added the following introductory line:

**Addition:** Results (Lines 181-182)

Table 1 shows the baseline characteristics of the patients in each group, and Table 2 lists the antibiotics included in each group.

***Comment 20***

*Line 174 & 175: consider adding significantly associated and not significantly associated.*

**Response 20:**

We have revised the relevant sentence according to your suggestion.

**Before:** Results (Lines 170-176)

In multivariate logistic regression analysis, older age (adjusted odds ratio [aOR], 1.06 [95% CI, 1.03–1.09], p < 0.001), higher CCI (aOR, 1.32 [95% CI, 1.19–1.47], p < 0.001), the presence of sepsis (aOR, 1.89 [95% CI, 1.17–3.06], p = 0.010), and ICU admission (aOR, 2.65 [95% CI, 1.26–5.58], p = 0.010) were associated with increased 30-day in-hospital mortality, whereas receipt of broad-spectrum empiric therapy was not (aOR, 1.37 [95% CI, 0.84–2.23], p = 0.20).

**After:** Results (Lines 205-211)

In multivariate logistic regression analysis, older age (adjusted odds ratio [aOR], 1.06 [95% CI, 1.03–1.09], p < 0.001), higher CCI (aOR, 1.32 [95% CI, 1.19–1.47], p < 0.001), the presence of sepsis (aOR, 1.89 [95% CI, 1.17–3.06], p = 0.010), and ICU admission (aOR, 2.65 [95% CI, 1.26–5.58], p = 0.010) were significantly associated with increased 30-day in-hospital mortality, whereas receipt of broad-spectrum empiric therapy was not significantly (aOR, 1.37 [95% CI, 0.84–2.23], p = 0.20).

***Comment 21***

*Table 3: Add* *significance level for p-value in footnotes, specify unit for age in the table,*

**Response 21:**

We have added the significance level (p = 0.05) to the footnote and clarified that age is expressed in years (y). Additionally, an error was identified in the notation of “Table 3,” and it has been corrected. We apologize for this oversight.

**Change:** Table 3

Please refer to Table 3.

***Comment 22***

*Line 187: consider adding not significantly associated.*

**Response 22:**

As you suggested, we have revised the relevant sentence.

**Before:** Results (Lines 186-188)

A conditional logistic regression model showed that receipt of broad-spectrum empiric therapy was not associated with 30-day in-hospital mortality (OR, 1.43 [95% CI, 0.82-2.50], p = 0.21).

**After:** Results (Lines 223-225)

A conditional logistic regression model showed that receipt of broad-spectrum empiric therapy was not significantly associated with 30-day in-hospital mortality (OR, 1.43 [95% CI, 0.82-2.50], p = 0.21).

***Comment 23***

*Line 189: “Table 4 presents the results regarding secondary outcomes” sentence is incomplete/unclear. Results of which test? Same for table caption*

**Response 23:**

Thank you for pointing this out. As suggested, we have revised the relevant sentence and Table 4.

**Before:** Results (Line 189)

Table 4 presents the results regarding secondary outcomes.

**After:** Results (Lines 228-229)

Table 4 presents the results regarding the incidence of CDI during hospitalization, duration of IV antibiotic therapy, and LOS.

**Before:** Table 4 caption

Table 4. Secondary outcomes in the PS-matched cohort

**After:** Table 4 caption

Table 4. Incidence of CDI, duration of IV antibiotic therapy, and LOS in the PS-matched cohort

***Comment 24***

*Table 4: Add significance level for p-value in footnotes,*

**Response 24:**

We have added the significance level (p = 0.05) to the footnote in Table 4.

**Change:** Table 4

Please refer to Table 4.

***Comment 25***

*Line 213: Was not associated with increased 30-day in hospital mortality,*

**Response 25:**

As suggested, we have revised the relevant sentence. To ensure consistency with other sentences, we have made the following changes:

**Before:** Discussion (Lines 212-213)

This study showed that administration of broad-spectrum empiric therapy was not associated with 30-day in-hospital mortality.

**After:** Discussion (Lines 254-255)

This study showed that administration of broad-spectrum empiric therapy was not associated with improved 30-day in-hospital mortality.

***Comment 26***

*Line 216: reference results table*

**Response 26:**

As you pointed out, we have added a reference to Table 4.

**Addition:** Discussion (Lines 258-259)

In addition, in the PS-matched cohort, patients in the broad-spectrum group had longer duration of IV antibiotic therapy and LOS (Table 4).

***Comment 27***

*Line 221: add citation*

**Response 27:**

Thank you for pointing this out. We have added citations to the relevant section and revised it for clarity.

**Before:** Discussion (Lines 220-227)

Broad-spectrum antibiotics are used against antimicrobial-resistant gram-negative pathogens. This study indicates that piperacillin/tazobactam and meropenem, which are generally active against third-generation cephalosporin-resistant *Enterobacterales* and *Pseudomonas aeruginosa*, are commonly used in broad-spectrum empiric therapy. In this study, blood culture results were unavailable because they were not included in the database. However, the prevalence of third-generation cephalosporin resistance among *E. coli* and *K. pneumoniae*, the main pathogens of acute cholangitis [1], is approximately 10–20% in Japan [11].

**After:** Discussion (Lines 263-268)

Piperacillin/tazobactam and meropenem, which are generally active against third-generation cephalosporin-resistant *Enterobacterales* and *P. aeruginosa* [31–33], were commonly used in broad-spectrum empiric therapy (Table 2). Although blood culture results were unavailable in this study, the prevalence of third-generation cephalosporin resistance among *E. coli* and *K. pneumoniae*, the main pathogens of acute cholangitis [1], is approximately 10–20% in Japan [32].

***Comment 28***

*Line 223: reference results table*

**Response 28:**

As you pointed out, we have added a reference to Table 2 in the relevant sentence of the revised manuscript.

**Before:** Discussion (Lines 221-223)

This study indicates that piperacillin/tazobactam and meropenem, which are generally active against third-generation cephalosporin-resistant *Enterobacterales* and *Pseudomonas aeruginosa*, are commonly used in broad-spectrum empiric therapy.

**After:** Discussion (Lines 263-265)

Piperacillin/tazobactam and meropenem, which are generally active against third-generation cephalosporin-resistant *Enterobacterales* and *P. aeruginosa* [31–33], were commonly used in broad-spectrum empiric therapy (Table 2).

***Comment 29***

*Line 230: 10 % may not be considered rare. Consider changing rare to lower or uncommon. Also add reference results table.*

**Response 29:**

As you suggested, we have revised the relevant sentence and added a reference to S1 Fig.

**Before:** Discussion (Lines 229-231)

In this study, escalation to broad-spectrum antibiotics as definitive therapy was rare in the narrow-spectrum group, indicating that most patients could be adequately treated with narrow-spectrum antibiotics.

**After:** Discussion (Lines 271-274)

Indeed, this study showed that escalation to broad-spectrum antibiotics as definitive therapy was uncommon in the narrow-spectrum group, suggesting that most patients were adequately treated with narrow-spectrum antibiotics (S1 Fig).

***Comment 30***

*Line 232: As the study results do not directly demonstrate low resistance, consider revising the wording to reflect a lower likelihood of resistant pathogens or a higher likelihood of pathogen susceptibility.*

**Response 30:**

As you suggested, we have revised the relevant sentence.

**Before:** Discussion (Lines 232-233)

In contrast, despite low resistance, 63.2% of the patients in the broad-spectrum group continued to receive broad-spectrum antibiotics without de-escalation.

**After:** Discussion (Lines 275-277)

In contrast, despite the lower likelihood of resistant pathogens in Japan, 63.2% of the patients in the broad-spectrum group continued to receive broad-spectrum antibiotics without de-escalation.

***Comment 31***

*Line 234- 238: The discussion suggests that broad-spectrum empiric therapy leads to unnecessary continuation of antibiotics based on negative blood cultures; however, this conclusion appears speculative and does not account for other factors influencing de-escalation decisions like limited microbiological data or CCI, sepsis, or ICU admissions.*

**Response 31:**

Thank you for your valuable comments. As you pointed out, the de-escalation and duration of IV antibiotic therapy may be influenced by various factors, including physician psychology and individual clinical backgrounds. To explain this point, we have revised the Discussion section as follows:

**Addition:** Discussion (Lines 296-299)

Nevertheless, de-escalation and the duration of IV antibiotic therapy may be influenced not only by the physician's psychology but also by patient-related factors, including individual clinical background [37,38]. Although this study adjusted for patient background using PS matching analysis, it could not fully account for these factors.

***Comment 32***

*Line 246: reference results table*

**Response 32:**

As you suggested, we have added a reference to Table 4 in the relevant sentence of the revised manuscript.

**Addition:** Discussion (Lines 286-287)

This study also showed that the duration of IV antibiotic therapy and LOS were longer in the broad-spectrum group versus the narrow-spectrum group (Table 4).

***Comment 33***

*Line 252: add citation*

**Response 33:**

The relevant sentence has been removed in accordance with the Reviewer 1’s comment 4. Thank you for pointing this out.

**Before:** Discussion (Lines 250-252)

However, the clinical impact of broad-spectrum empiric therapy on patient outcomes may vary depending on regional resistance rates.

**After:** [Deletion]

***Comment 34***

*Line 260: Add Implication of this limitation on study results*

**Response 34:**

As you pointed out, we have added an explanation of the impact of this limitation on the results.

**Addition:** Discussion (Lines 309-310)

Therefore, residual confounding may exist based on the indications and severity.

***Comment 35***

*The repeated characterization of broad-spectrum antibiotic use as “unnecessary” in the Discussion and Conclusion may overstate the findings. Consider revising the wording to use more neutral terms that better reflect the observational nature of the study (suggestions: avoid prolonged use or cautious/ culture guided use is warranted).*

**Response 35:**

Thank you for this helpful suggestion. Considering the limitations of this study, we have revised the sentence containing the word “unnecessary” to a more neutral expression as follows. Furthermore, throughout the discussion, we have revised the text to present arguments from a more neutral perspective. We have also clearly stated in the Discussion section that our results do not imply that broad-spectrum empiric therapy for acute cholangitis is always unnecessary.

**Before:** Discussion (Lines 228-231)

Therefore, broad-spectrum empiric therapy is unnecessary in most patients with acute cholangitis. In this study, escalation to broad-spectrum antibiotics as definitive therapy was rare in the narrow-spectrum group, indicating that most patients could be adequately treated with narrow-spectrum antibiotics.

**After:** Discussion (Lines 269-274)

These epidemiological data suggest that a large proportion of patients with acute cholangitis in Japan can be treated without broad-spectrum antibiotics. Indeed, this study showed that escalation to broad-spectrum antibiotics as definitive therapy was uncommon in the narrow-spectrum group, suggesting that most patients were adequately treated with narrow-spectrum antibiotics (S1 Fig).

**Before:** Discussion (Lines 237-238)

Thus, broad-spectrum empiric therapy may lead to unnecessary continuation of broad-spectrum antibiotics.

**After:** Discussion (Lines 277-279)

This finding implies that de-escalation may not be sufficiently implemented in patients with acute cholangitis who received broad-spectrum empiric therapy.

**Before:** Discussion (Lines 241-243)

Previous studies have reported that unnecessary use of broad-spectrum antibiotic is associated with increased mortality, increased incidence of CDI, and prolonged LOS [13–15].

**After:** [Deletion]

Following this deletion, we have revised the two sentences below in the relevant section of the Discussion.

**Before:** Discussion (Line 245-246)

In contrast, the duration of IV antibiotic therapy and LOS were longer in the broad-spectrum group versus the narrow-spectrum group.

**After:** Discussion (Lines 286-287)

This study also showed that the duration of IV antibiotic therapy and LOS were longer in the broad-spectrum group versus the narrow-spectrum group (Table 4).

**Before:** Discussion (Line 243)

In this study, the incidence of CDI did not significantly differ between the groups.

**After:** Discussion (Lines 292-294)

Although broad-spectrum empiric therapy has been reported to be associated with an increased incidence of CDI [11,16], no significant difference was observed between the groups in this study (Table 4).

**Before:** Discussion (Line 248-250)

These findings suggest that unnecessary broad-spectrum empiric therapy may be associated with prolonged IV antibiotic therapy and LOS in patients with acute cholangitis.

**After:** Discussion (Lines 290-292)

These findings suggest that measures are needed to avoid unnecessary prolonged use of antibiotics, particularly in patients who have received broad-spectrum empiric therapy.

**Addition:** Discussion (Lines 300-303)

Therefore, our findings do not imply that broad-spectrum empiric therapy is always unnecessary for the treatment of acute cholangitis. Rather, these results suggest that broad-spectrum empiric therapy for acute cholangitis could be an important focus of antimicrobial stewardship, highlighting the need for more judicious indications.

**Before:** Conclusion (Lines 274-276)

The active use of narrow-spectrum antibiotics as empiric therapy can help reduce the unnecessary use of broad-spectrum antibiotics and may contribute to the prevention of antimicrobial resistance.

**After:** Conclusion (Lines 330-332)

The active use of narrow-spectrum antibiotics as empiric therapy can help reduce the use of broad-spectrum antibiotics and may contribute to the prevention of antimicrobial resistance.
